# Supplementary material for: The ameliorative effect of pioglitazone against colistin-induced nephrotoxicity is mediated by inhibition of NF-κB and restoration of Nrf2 signaling: An integrative bioinformatics prediction-guided in vitro study
Source: PLoS One. 2024 Dec 2;19(12):e0314092. doi: 10.1371/journal.pone.0314092 (PMC11611169; doi:10.1371/journal.pone.0314092)
Supplement: S1 File — (DOCX) [file pone.0314092.s001.docx]

The Ameliorative Effect of Pioglitazone Against Colistin-induced Nephrotoxicity is Mediated by Inhibition of NF-κB and Restoration of Nrf2 Signaling: An Integrative Bioinformatics Prediction-Guided In Vitro Study

Metab Alharbi ^1 *^ † , Mohamed A. Mahmoud ^1^ †_,_ Abdulrahman Alshammari ^1^, Mashal M. Almutairi ^1^, Jihan M. Al-Ghamdi ^2^, Jawza F. Alsabhan ^3^, Othman Al Shabanah ^1^, Norah . A . Alshalawi ^1^, Sami I. Alzarea ^4^ and Abdullah F. Alasmari ^1^

^1 Department of Pharmacology and Toxicology, College of Pharmacy, King Saud University, P.O. Box 2457, Riyadh 11451, Saudi Arabia^

^2 Biochemistry Department, College of Science, King Saud University, P.O. Box. 2460, Riyadh 11451, Saudi Arabia^

^3 Department of clinical pharmacy, college of pharmacy, King Saud University, Riyadh, Saudi Arabia^

^4 Department of Pharmacology, College of Pharmacy, Jouf University, Sakaka, Aljouf 72341, Saudi Arabia^

^† These authors contributed equally to this work^

^* Correspondence: mesalharbi@ksu.edu.sa^

**Table S1.** Acute kidney injury-related genes.

| **Gene** | **Gene ID** | **Gene Full Name** |
| --- | --- | --- |

| SIRT1 | 23411 | sirtuin 1 |
| --- | --- | --- |
| PPARG | 5468 | peroxisome proliferator activated receptor gamma |
| CLU | 1191 | clusterin |
| HMOX1 | 3162 | heme oxygenase 1 |
| HP | 3240 | haptoglobin |
| B2M | 567 | beta-2-microglobulin |
| HBEGF | 1839 | heparin binding EGF like growth factor |
| GFER | 2671 | growth factor, augmenter of liver regeneration |
| NQO1 | 1728 | NAD(P)H quinone dehydrogenase 1 |
| TLR4 | 7099 | toll like receptor 4 |
| HAVCR1 | 26762 | hepatitis A virus cellular receptor 1 |
| BAX | 581 | BCL2 associated X, apoptosis regulator |
| ATP5F1B | 506 | ATP synthase F1 subunit beta |
| NOS3 | 4846 | nitric oxide synthase 3 |
| NPPA | 4878 | natriuretic peptide A |
| NFE2L2 | 4780 | nuclear factor, erythroid 2 like 2 |
| LCN2 | 3934 | lipocalin 2 |
| MPO | 4353 | myeloperoxidase |
| EGFR | 1956 | epidermal growth factor receptor |
| C3 | 718 | complement C3 |
| SLC22A12 | 116085 | solute carrier family 22 member 12 |
| UGT1A9 | 54600 | UDP glucuronosyltransferase family 1 member A9 |
| IGFBP1 | 3484 | insulin like growth factor binding protein 1 |
| UGT1A1 | 54658 | UDP glucuronosyltransferase family 1 member A1 |
| INS | 3630 | insulin |
| IL6 | 3569 | interleukin 6 |
| IL10 | 3586 | interleukin 10 |
| IGF1 | 3479 | insulin like growth factor 1 |
| GSTP1 | 2950 | glutathione S-transferase pi 1 |
| MIR146B | 574447 | microRNA 146b |
| HBG2 | 3048 | hemoglobin subunit gamma 2 |
| HEXB | 3074 | hexosaminidase subunit beta |
| MTHFR | 4524 | methylenetetrahydrofolate reductase |
| IFNA2 | 3440 | interferon alpha 2 |
| HPX | 3263 | hemopexin |
| HSPA1A | 3303 | heat shock protein family A (Hsp70) member 1A |
| AVP | 551 | arginine vasopressin |
| KLK1 | 3816 | kallikrein 1 |
| KNG1 | 3827 | kininogen 1 |
| MIR25 | 407014 | microRNA 25 |
| PLG | 5340 | plasminogen |
| MIR30A | 407029 | microRNA 30a |
| MIR30D | 407033 | microRNA 30d |
| MIR99A | 407055 | microRNA 99a |
| SERPINA1 | 5265 | serpin family A member 1 |
| TNFRSF12A | 51330 | TNF receptor superfamily member 12A |
| IL20 | 50604 | interleukin 20 |
| NFKB1 | 4790 | nuclear factor kappa B subunit 1 |
| NOS2 | 4843 | nitric oxide synthase 2 |
| MIR23A | 407010 | microRNA 23a |
| MIR215 | 406997 | microRNA 215 |
| POMC | 5443 | proopiomelanocortin |
| UOX | 391051 | urate oxidase (pseudogene) |
| LRP2 | 4036 | LDL receptor related protein 2 |
| MIRLET7I | 406891 | microRNA let-7i |
| MIR10A | 406902 | microRNA 10a |
| MIR122 | 406906 | microRNA 122 |
| MIR140 | 406932 | microRNA 140 |
| MIR143 | 406935 | microRNA 143 |
| MIR148A | 406940 | microRNA 148a |
| MIR192 | 406967 | microRNA 192 |
| ORM1 | 5004 | orosomucoid 1 |
| GSTM2 | 2946 | glutathione S-transferase mu 2 |
| RAPGEF3 | 10411 | Rap guanine nucleotide exchange factor 3 |
| CD44 | 960 | CD44 molecule (Indian blood group) |
| TNF | 7124 | tumor necrosis factor |
| TLR2 | 7097 | toll like receptor 2 |
| AFM | 173 | afamin |
| THBD | 7056 | thrombomodulin |
| EDN1 | 1906 | endothelin 1 |
| TGFB1 | 7040 | transforming growth factor beta 1 |
| AHSG | 197 | alpha 2-HS glycoprotein |
| A2M | 2 | alpha-2-macroglobulin |
| EPHX2 | 2053 | epoxide hydrolase 2 |
| CYP2D6 | 1565 | cytochrome P450 family 2 subfamily D member 6 |
| CYP2C9 | 1559 | cytochrome P450 family 2 subfamily C member 9 |
| CYP2C19 | 1557 | cytochrome P450 family 2 subfamily C member 19 |
| GPNMB | 10457 | glycoprotein nmb |
| AMN | 81693 | amnion associated transmembrane protein |
| OCLN | 100506658 | occludin |
| VEGFA | 7422 | vascular endothelial growth factor A |
| FSTL1 | 11167 | follistatin like 1 |
| TTR | 7276 | transthyretin |
| CCR5 | 1234 | C-C motif chemokine receptor 5 (gene/pseudogene) |
| CP | 1356 | ceruloplasmin |
| CST3 | 1471 | cystatin C |
| TP53 | 7157 | tumor protein p53 |
| EPO | 2056 | erythropoietin |
| TFF3 | 7033 | trefoil factor 3 |
| ALB | 213 | albumin |
| GC | 2638 | GC vitamin D binding protein |
| GAS6 | 2621 | growth arrest specific 6 |
| AMBP | 259 | alpha-1-microglobulin/bikunin precursor |
| G6PD | 2539 | glucose-6-phosphate dehydrogenase |
| SLC22A24 | 283238 | solute carrier family 22 member 24 |
| BDKRB2 | 624 | bradykinin receptor B2 |
| SLC11A1 | 6556 | solute carrier family 11 member 1 |
| TF | 7018 | transferrin |
| SOD1 | 6647 | superoxide dismutase 1 |
| GSK3B | 2932 | glycogen synthase kinase 3 beta |
| SPP1 | 6696 | secreted phosphoprotein 1 |
| GSTA1 | 2938 | glutathione S-transferase alpha 1 |
| CAPN1 | 823 | calpain 1 |
| PTGER4 | 5734 | prostaglandin E receptor 4 |
| SCGB1A1 | 7356 | secretoglobin family 1A member 1 |
| PTGS2 | 5743 | prostaglandin-endoperoxide synthase 2 |
| HDAC3 | 8841 | histone deacetylase 3 |
| CASP1 | 834 | caspase 1 |
| FIS1 | 51024 | fission, mitochondrial 1 |
| RB1 | 5925 | RB transcriptional corepressor 1 |
| PKD2 | 5311 | polycystin 2, transient receptor potential cation channel |
| BCL2 | 596 | BCL2 apoptosis regulator |
| EGLN1 | 54583 | egl-9 family hypoxia inducible factor 1 |
| TGFA | 7039 | transforming growth factor alpha |
| CYP2E1 | 1571 | cytochrome P450 family 2 subfamily E member 1 |
| WNT4 | 54361 | Wnt family member 4 |
| SLC22A2 | 6582 | solute carrier family 22 member 2 |
| SLC22A1 | 6580 | solute carrier family 22 member 1 |
| AVPR1A | 552 | arginine vasopressin receptor 1A |
| MFN1 | 55669 | mitofusin 1 |
| BAD | 572 | BCL2 associated agonist of cell death |
| BCL2L1 | 598 | BCL2 like 1 |
| PTAFR | 5724 | platelet activating factor receptor |
| XDH | 7498 | xanthine dehydrogenase |
| CDKN1B | 1027 | cyclin dependent kinase inhibitor 1B |
| ALAD | 210 | aminolevulinate dehydratase |
| FN1 | 2335 | fibronectin 1 |
| HDAC5 | 10014 | histone deacetylase 5 |
| ALOX5 | 240 | arachidonate 5-lipoxygenase |
| FGA | 2243 | fibrinogen alpha chain |
| MTOR | 2475 | mechanistic target of rapamycin kinase |
| GATM | 2628 | glycine amidinotransferase |
| GOT2 | 2806 | glutamic-oxaloacetic transaminase 2 |
| UTS2R | 2837 | urotensin 2 receptor |
| EPAS1 | 2034 | endothelial PAS domain protein 1 |
| EGF | 1950 | epidermal growth factor |
| PROCR | 10544 | protein C receptor |
| NES | 10763 | nestin |
| EGLN3 | 112399 | egl-9 family hypoxia inducible factor 3 |
| CRP | 1401 | C-reactive protein |
| ADRB2 | 154 | adrenoceptor beta 2 |
| CYP27B1 | 1594 | cytochrome P450 family 27 subfamily B member 1 |
| ACE | 1636 | angiotensin I converting enzyme |
| DDIT3 | 1649 | DNA damage inducible transcript 3 |
| ECE1 | 1889 | endothelin converting enzyme 1 |
| GSR | 2936 | glutathione-disulfide reductase |
| ANXA1 | 301 | annexin A1 |
| ARG1 | 383 | arginase 1 |
| NDUFB8 | 4714 | NADH:ubiquinone oxidoreductase subunit B8 |
| HIF1A | 3091 | hypoxia inducible factor 1 subunit alpha |
| MET | 4233 | MET proto-oncogene, receptor tyrosine kinase |
| IL1B | 3553 | interleukin 1 beta |
| AR | 367 | androgen receptor |
| ARG2 | 384 | arginase 2 |
| LGALS3 | 3958 | galectin 3 |
| MIR26A1 | 407015 | microRNA 26a-1 |
| IGF1 | 3480 | insulin like growth factor 1 receptor |
| MB | 4151 | myoglobin |
| NPPB | 4879 | natriuretic peptide B |
| NOS1 | 4842 | nitric oxide synthase 1 |
| HES1 | 3280 | hes family bHLH transcription factor 1 |
| HSPA8 | 3312 | heat shock protein family A (Hsp70) member 8 |
| ICAM1 | 3383 | intercellular adhesion molecule 1 |
| CFHR3 | 10878 | complement factor H related 3 |
| CORIN | 10699 | corin, serine peptidase |
| SLC26A1 | 10861 | solute carrier family 26 member 1 |
| CACNA1S | 779 | calcium voltage-gated channel subunit alpha1 S |
| CD46 | 4179 | CD46 molecule |
| DGKE | 8526 | diacylglycerol kinase epsilon |
| HELLPAR | 101101692 | HELLP associated long non-coding RNA |
| TRIM25 | 7706 | tripartite motif containing 25 |
| CFH | 3075 | complement factor H |
| CFHR1 | 3078 | complement factor H related 1 |
| CCND1 | 595 | cyclin D1 |
| HNF4A | 3172 | hepatocyte nuclear factor 4 alpha |
| HPRT1 | 3251 | hypoxanthine phosphoribosyltransferase 1 |
| RYR1 | 6261 | ryanodine receptor 1 |
| CFB | 629 | complement factor B |
| CFI | 3426 | complement factor I |
| FLT1 | 2321 | fms related receptor tyrosine kinase 1 |
| LPIN1 | 23175 | lipin 1 |
| SAA1 | 6288 | serum amyloid A1 |
| TCN2 | 6948 | transcobalamin 2 |
| STOX1 | 219736 | storkhead box 1 |
| HNF1B | 6928 | HNF1 homeobox B |

**Table S2.** Pioglitazone-related genes.

| **Gene** | **Gene ID** | **Interaction Count** |
| --- | --- | --- |

| PPARG | 5468 | 106 |
| --- | --- | --- |
| TNF | 7124 | 27 |
| ADIPOQ | 9370 | 20 |
| INS | 3630 | 19 |
| IL1B | 3553 | 16 |
| PPARA | 5465 | 13 |
| RELA | 5970 | 13 |
| CYP3A4 | 1576 | 11 |
| IL6 | 3569 | 11 |
| CD36 | 948 | 10 |
| CDKN1A | 1026 | 10 |
| FABP4 | 2167 | 10 |
| PDGFB | 5155 | 9 |
| PRNP | 5621 | 9 |
| ABCA1 | 19 | 8 |
| NR1H3 | 10062 | 7 |
| BACE1 | 23621 | 6 |
| BSG | 682 | 6 |
| CAT | 847 | 6 |
| INS1 | 16333 | 6 |
| MAPK1 | 5594 | 6 |
| TGFB1 | 7040 | 6 |
| CASP3 | 836 | 5 |
| CCND1 | 595 | 5 |
| CDKN1B | 1027 | 5 |
| CYP17A1 | 1586 | 5 |
| NOS2 | 4843 | 5 |
| NPHS1 | 4868 | 5 |
| NQO1 | 1728 | 5 |
| PCSK9 | 255738 | 5 |
| PPARD | 5467 | 5 |
| RETN | 56729 | 5 |
| RGN | 9104 | 5 |
| VEGFA | 7422 | 5 |
| ACTA2 | 59 | 4 |
| AGT | 183 | 4 |
| APP | 351 | 4 |
| CYP2C8 | 1558 | 4 |
| DIO2 | 1734 | 4 |
| HMOX1 | 3162 | 4 |
| MAPK3 | 5595 | 4 |
| MMP9 | 4318 | 4 |
| NFE2L2 | 4780 | 4 |
| PTGS2 | 5743 | 4 |
| RPS6 | 6194 | 4 |
| ABCB11 | 8647 | 3 |
| ACE2 | 59272 | 3 |
| CCL2 | 6347 | 3 |
| CCN2 | 1490 | 3 |
| CXCL12 | 6387 | 3 |
| CYP7A1 | 1581 | 3 |
| EDN1 | 1906 | 3 |
| EIF4EBP1 | 1978 | 3 |
| HSD3B2 | 3284 | 3 |
| IFNG | 3458 | 3 |
| IL18 | 3606 | 3 |
| LEPR | 3953 | 3 |
| PRKAA1 | 5562 | 3 |
| RETSAT | 54884 | 3 |
| RPS6KB1 | 6198 | 3 |
| RXRA | 6256 | 3 |
| S100A8 | 6279 | 3 |
| S100A9 | 6280 | 3 |
| SCARB1 | 949 | 3 |
| SOD1 | 6647 | 3 |
| VCAM1 | 7412 | 3 |
| ACE | 1636 | 2 |
| AGTR2 | 186 | 2 |
| ANGPTL2 | 23452 | 2 |
| ANKRD37 | 353322 | 2 |
| APOA1 | 335 | 2 |
| APOC2 | 344 | 2 |
| BAX | 581 | 2 |
| BCL2 | 596 | 2 |
| BZW1 | 9689 | 2 |
| CCL3 | 6348 | 2 |
| CCL5 | 6352 | 2 |
| CRP | 1401 | 2 |
| CRYAB | 1410 | 2 |
| CXCL2 | 2920 | 2 |
| CXCL5 | 6374 | 2 |
| CXCL8 | 3576 | 2 |
| CYBA | 1535 | 2 |
| DDIT4 | 54541 | 2 |
| DDIT4L | 115265 | 2 |
| DEPP1 | 11067 | 2 |
| DNAAF2 | 55172 | 2 |
| FGF21 | 26291 | 2 |
| GPT | 2875 | 2 |
| HIF1A | 3091 | 2 |
| HILPDA | 29923 | 2 |
| HMGCR | 3156 | 2 |
| HNRNPDL | 9987 | 2 |
| HSPA1L | 3305 | 2 |
| ID2 | 3398 | 2 |
| IGF1 | 3479 | 2 |
| IL10 | 3586 | 2 |
| IL24 | 11009 | 2 |
| KLF2 | 10365 | 2 |
| KLF5 | 688 | 2 |
| LDLR | 3949 | 2 |
| LIPG | 9388 | 2 |
| LITAF | 9516 | 2 |
| MMP7 | 4316 | 2 |
| MXD1 | 4084 | 2 |
| PIK3CA | 5290 | 2 |
| PLD1 | 5337 | 2 |
| PLIN1 | 5346 | 2 |
| PLIN2 | 123 | 2 |
| PPARGC1A | 10891 | 2 |
| PRKCA | 5578 | 2 |
| RICTOR | 253260 | 2 |
| RPS6KA2 | 6196 | 2 |
| RPTOR | 57521 | 2 |
| SCNN1G | 6340 | 2 |
| SERPINE1 | 5054 | 2 |
| SLC2A5 | 6518 | 2 |
| SPP1 | 6696 | 2 |
| ST3GAL1 | 6482 | 2 |
| TAGLN | 6876 | 2 |
| TELO2 | 9894 | 2 |
| THBS1 | 7057 | 2 |
| TRIB3 | 57761 | 2 |
| ULK1 | 8408 | 2 |
| VASN | 114990 | 2 |
| VLDLR | 7436 | 2 |
| ABAT | 18 | 1 |
| ABCA4 | 24 | 1 |
| ABCA6 | 23460 | 1 |
| ABCA8A | 217258 | 1 |
| ABCA8B | 27404 | 1 |
| ABCB1 | 5243 | 1 |
| ABCC9 | 10060 | 1 |
| ABI3BP | 25890 | 1 |
| ABLIM2 | 84448 | 1 |
| ABRA | 137735 | 1 |
| ACAT1 | 38 | 1 |
| ACAT2 | 39 | 1 |
| ACOD1 | 730249 | 1 |
| ACTA1 | 58 | 1 |
| ACTC1 | 70 | 1 |
| ACTG2 | 72 | 1 |
| ACTN2 | 88 | 1 |
| ADAM23 | 8745 | 1 |
| ADAM5 | 255926 | 1 |
| ADAM9 | 8754 | 1 |
| ADCY5 | 111 | 1 |
| ADCYAP1R1 | 117 | 1 |
| ADHFE1 | 137872 | 1 |
| ADM | 133 | 1 |
| ADORA1 | 134 | 1 |
| AGBL1 | 123624 | 1 |
| AGER | 177 | 1 |
| AGTR1A | 11607 | 1 |
| AIFM3 | 150209 | 1 |
| AJUBA | 84962 | 1 |
| ALPK3 | 57538 | 1 |
| AMIGO2 | 347902 | 1 |
| AMOTL2 | 51421 | 1 |
| AMPD1 | 270 | 1 |
| ANGPT2 | 285 | 1 |
| ANGPTL4 | 51129 | 1 |
| ANK1 | 286 | 1 |
| ANKRD63 | 100131244 | 1 |
| ANO5 | 203859 | 1 |
| AOC3 | 8639 | 1 |
| AP1S2 | 8905 | 1 |
| APC2 | 10297 | 1 |
| APOB | 338 | 1 |
| APOC1 | 341 | 1 |
| APOC3 | 345 | 1 |
| APOL6 | 80830 | 1 |
| AQP7 | 364 | 1 |
| AR | 367 | 1 |
| AREG | 374 | 1 |
| ARMCX2 | 9823 | 1 |
| ART1 | 417 | 1 |
| ART2A | 11871 | 1 |
| ASB10 | 136371 | 1 |
| ASB14 | 142686 | 1 |
| ASB5 | 140458 | 1 |
| ATF3 | 467 | 1 |
| ATP1A2 | 477 | 1 |
| ATP2A1 | 487 | 1 |
| ATP5F1B | 506 | 1 |
| AVPR1A | 552 | 1 |
| B4GALNT2 | 124872 | 1 |
| B4GALNT3 | 283358 | 1 |
| BCHE | 590 | 1 |
| BCL11A | 53335 | 1 |
| BDNF | 627 | 1 |
| BEND5 | 79656 | 1 |
| BGLAP | 632 | 1 |
| BHLHA15 | 168620 | 1 |
| BIRC3 | 330 | 1 |
| BMP6 | 654 | 1 |
| BPIFA5 | 67135 | 1 |
| BPIFB1 | 92747 | 1 |
| BTN2A2 | 10385 | 1 |
| C1ORF54 | 79630 | 1 |
| C1QTNF7 | 114905 | 1 |
| C4A | 720 | 1 |
| C5AR1 | 728 | 1 |
| CABCOCO1 | 219621 | 1 |
| CACNA1C | 775 | 1 |
| CACNA1S | 779 | 1 |
| CACNA2D3 | 55799 | 1 |
| CADM2 | 253559 | 1 |
| CALHM5 | 254228 | 1 |
| CAR3 | 12350 | 1 |
| CASQ1 | 844 | 1 |
| CAV1 | 857 | 1 |
| CAV3 | 859 | 1 |
| CCDC159 | 126075 | 1 |
| CCDC85A | 114800 | 1 |
| CCL11 | 6356 | 1 |
| CCL3L1 | 6349 | 1 |
| CCL3L3 | 414062 | 1 |
| CCL7 | 6354 | 1 |
| CCNA1 | 8900 | 1 |
| CCNA2 | 890 | 1 |
| CCNB1 | 891 | 1 |
| CCND3 | 896 | 1 |
| CCNE1 | 898 | 1 |
| CCNO | 10309 | 1 |
| CD209F | 69142 | 1 |
| CD84 | 8832 | 1 |
| CDC20B | 166979 | 1 |
| CDC42 | 998 | 1 |
| CDK2 | 1017 | 1 |
| CDKL1 | 8814 | 1 |
| CDKN1C | 1028 | 1 |
| CEBPA | 1050 | 1 |
| CEMIP | 57214 | 1 |
| CES1 | 1066 | 1 |
| CES1C | 13884 | 1 |
| CFD | 1675 | 1 |
| CGB1 | 114335 | 1 |
| CGB5 | 93659 | 1 |
| CHCHD6 | 84303 | 1 |
| CHI3L1 | 1116 | 1 |
| CHIT1 | 1118 | 1 |
| CHP2 | 63928 | 1 |
| CHRM1 | 1128 | 1 |
| CHRM2 | 1129 | 1 |
| CHRNA1 | 1134 | 1 |
| CHRNE | 1145 | 1 |
| CHUK | 1147 | 1 |
| CIDEC | 63924 | 1 |
| CKM | 1158 | 1 |
| CKMT2 | 1160 | 1 |
| CLDN3 | 1365 | 1 |
| CLDN4 | 1364 | 1 |
| CLEC14A | 161198 | 1 |
| CLEC1A | 51267 | 1 |
| CLEC4A | 50856 | 1 |
| CLEC4D | 338339 | 1 |
| CLEC4E | 26253 | 1 |
| CLSTN3 | 9746 | 1 |
| CMYA5 | 202333 | 1 |
| CNGA3 | 1261 | 1 |
| CNN1 | 1264 | 1 |
| CNRIP1 | 25927 | 1 |
| CNTD1 | 124817 | 1 |
| COG8 | 84342 | 1 |
| COL6A5 | 256076 | 1 |
| CORO6 | 84940 | 1 |
| COX6A2 | 1339 | 1 |
| COX8B | 12869 | 1 |
| CPT1A | 1374 | 1 |
| CPT1B | 1375 | 1 |
| CPXM1 | 56265 | 1 |
| CPZ | 8532 | 1 |
| CREB1 | 1385 | 1 |
| CRH | 1392 | 1 |
| CRHR2 | 1395 | 1 |
| CRISPLD1 | 83690 | 1 |
| CRYM | 1428 | 1 |
| CSHL1 | 1444 | 1 |
| CSN1S1 | 1446 | 1 |
| CSRP3 | 8048 | 1 |
| CTLA4 | 1493 | 1 |
| CTNNB1 | 1499 | 1 |
| CXCL15 | 20309 | 1 |
| CXCL3 | 2921 | 1 |
| CXCR3 | 2833 | 1 |
| CXCR4 | 7852 | 1 |
| CYP2B1 | 24300 | 1 |
| CYP2B10 | 13088 | 1 |
| CYP2B6 | 1555 | 1 |
| CYP2C55 | 72082 | 1 |
| CYP2E1 | 1571 | 1 |
| CYP2G1 | 13108 | 1 |
| CYP3A2 | 266682 | 1 |
| CYP3A23-3A1 | 25642 | 1 |
| CYP4A12A | 277753 | 1 |
| CYP4F40 | 631304 | 1 |
| DCAF12L1 | 139170 | 1 |
| DCPP1 | 13184 | 1 |
| DCPP2 | 630537 | 1 |
| DCPP3 | 620253 | 1 |
| DCX | 1641 | 1 |
| DDAH1 | 23576 | 1 |
| DEFB3 | 27358 | 1 |
| DEPTOR | 64798 | 1 |
| DERL3 | 91319 | 1 |
| DES | 1674 | 1 |
| DEUP1 | 159989 | 1 |
| DGAT1 | 8694 | 1 |
| DGAT2 | 84649 | 1 |
| DHRS7C | 201140 | 1 |
| DIO3 | 1735 | 1 |
| DLG1 | 1739 | 1 |
| DMBT1 | 1755 | 1 |
| DNAH8 | 1769 | 1 |
| DNER | 92737 | 1 |
| DNMT3AOS | 100038522 | 1 |
| DPT | 1805 | 1 |
| DPY19L2 | 283417 | 1 |
| DRD1 | 1812 | 1 |
| DUSP10 | 11221 | 1 |
| DUSP26 | 78986 | 1 |
| EDDM3B | 64184 | 1 |
| EEF1A2 | 1917 | 1 |
| EGF | 1950 | 1 |
| EGFLAM | 133584 | 1 |
| EIF4E | 1977 | 1 |
| EIF4EBP2 | 1979 | 1 |
| EIF5A | 1984 | 1 |
| ELOVL4 | 6785 | 1 |
| ENO3 | 2027 | 1 |
| EPGN | 255324 | 1 |
| EPHA3 | 2042 | 1 |
| EPHA5 | 2044 | 1 |
| EPYC | 1833 | 1 |
| ERBIN | 55914 | 1 |
| EREG | 2069 | 1 |
| ERVMER34-1 | 100288413 | 1 |
| F10 | 2159 | 1 |
| FABP3 | 2170 | 1 |
| FAM13A | 10144 | 1 |
| FAM171B | 165215 | 1 |
| FAM184B | 27146 | 1 |
| FAM210A | 125228 | 1 |
| FAM240B | 110806297 | 1 |
| FAM47E | 100129583 | 1 |
| FAT4 | 79633 | 1 |
| FBLN1 | 2192 | 1 |
| FBLN7 | 129804 | 1 |
| FBXL16 | 146330 | 1 |
| FBXO40 | 51725 | 1 |
| FBXO43 | 286151 | 1 |
| FCMR | 9214 | 1 |
| FGF7 | 2252 | 1 |
| FGL1 | 2267 | 1 |
| FHL1 | 2273 | 1 |
| FILIP1 | 27145 | 1 |
| FJX1 | 24147 | 1 |
| FKBP1A | 2280 | 1 |
| FKBP8 | 23770 | 1 |
| FLNC | 2318 | 1 |
| FNDC5 | 252995 | 1 |
| FOS | 2353 | 1 |
| FOSB | 2354 | 1 |
| FOSL1 | 8061 | 1 |
| FOXN4 | 121643 | 1 |
| FPR1 | 2357 | 1 |
| FRZB | 2487 | 1 |
| FSCN1 | 6624 | 1 |
| FSD2 | 123722 | 1 |
| FXYD1 | 5348 | 1 |
| GABRA3 | 2556 | 1 |
| GABRA4 | 2557 | 1 |
| GALNT16 | 57452 | 1 |
| GALNT17 | 64409 | 1 |
| GALNT9 | 50614 | 1 |
| GBP10 | 626578 | 1 |
| GDF15 | 9518 | 1 |
| GEM | 2669 | 1 |
| GEMIN4 | 50628 | 1 |
| GFRA1 | 2674 | 1 |
| GH1 | 2688 | 1 |
| GIMAP4 | 55303 | 1 |
| GLDN | 342035 | 1 |
| GLYCAM1 | 644076 | 1 |
| GMNC | 647309 | 1 |
| GNAT3 | 346562 | 1 |
| GOT1 | 2805 | 1 |
| GOT1L1 | 137362 | 1 |
| GP2 | 2813 | 1 |
| GPIHBP1 | 338328 | 1 |
| GPM6A | 2823 | 1 |
| GPR183 | 1880 | 1 |
| GPR84 | 53831 | 1 |
| GPRASP2 | 114928 | 1 |
| GPX3 | 2878 | 1 |
| GRM4 | 2914 | 1 |
| GSK3B | 2932 | 1 |
| GSTA2 | 2939 | 1 |
| GSTP1 | 2950 | 1 |
| GSTT1 | 2952 | 1 |
| GUCY1A1 | 2982 | 1 |
| HBB | 3043 | 1 |
| HECW1 | 23072 | 1 |
| HHATL | 57467 | 1 |
| HHIP | 64399 | 1 |
| HIF3A | 64344 | 1 |
| HJV | 148738 | 1 |
| HMGCS1 | 3157 | 1 |
| HPGD | 3248 | 1 |
| HPSE2 | 60495 | 1 |
| HRC | 3270 | 1 |
| HSPA12B | 116835 | 1 |
| HSPB7 | 27129 | 1 |
| HTR2B | 3357 | 1 |
| ICAM1 | 3383 | 1 |
| ICAM3 | 3385 | 1 |
| IFITM6 | 213002 | 1 |
| IGSF10 | 285313 | 1 |
| IKBKB | 3551 | 1 |
| IL13 | 3596 | 1 |
| IL1A | 3552 | 1 |
| IL23A | 51561 | 1 |
| IL4 | 3565 | 1 |
| IL5 | 3567 | 1 |
| ILK | 3611 | 1 |
| INMT | 11185 | 1 |
| INSYN2B | 100131897 | 1 |
| IQCH | 64799 | 1 |
| IRAG1 | 10335 | 1 |
| ISL1 | 3670 | 1 |
| ISM2 | 145501 | 1 |
| ITGA8 | 8516 | 1 |
| ITGAV | 3685 | 1 |
| ITGB1BP2 | 26548 | 1 |
| ITIH2 | 3698 | 1 |
| ITLN1 | 55600 | 1 |
| JCHAIN | 3512 | 1 |
| JPH2 | 57158 | 1 |
| JSRP1 | 126306 | 1 |
| KANK4 | 163782 | 1 |
| KCNB1 | 3745 | 1 |
| KCNC4 | 3749 | 1 |
| KCND3 | 3752 | 1 |
| KCNJ11 | 3767 | 1 |
| KCNJ13 | 3769 | 1 |
| KLHL13 | 90293 | 1 |
| KLHL31 | 401265 | 1 |
| KLHL40 | 131377 | 1 |
| KLHL41 | 10324 | 1 |
| KLK6 | 5653 | 1 |
| KRT16 | 3868 | 1 |
| KRT20 | 54474 | 1 |
| KRT42 | 68239 | 1 |
| KRT6A | 3853 | 1 |
| KRT6B | 3854 | 1 |
| KRT84 | 3890 | 1 |
| KRT90 | 239673 | 1 |
| KY | 339855 | 1 |
| LAMA2 | 3908 | 1 |
| LARP6 | 55323 | 1 |
| LCE1F | 353137 | 1 |
| LCE1I | 76585 | 1 |
| LEP | 3952 | 1 |
| LGI1 | 9211 | 1 |
| LHFPL4 | 375323 | 1 |
| LILRA2 | 11027 | 1 |
| LIPC | 3990 | 1 |
| LIPF | 8513 | 1 |
| LIPN | 643418 | 1 |
| LMAN1L | 79748 | 1 |
| LMOD3 | 56203 | 1 |
| LRRC39 | 127495 | 1 |
| LRRC3B | 116135 | 1 |
| LRRN1 | 57633 | 1 |
| LRRN3 | 54674 | 1 |
| LYZ1 | 17110 | 1 |
| MAGIX | 79917 | 1 |
| MAOB | 4129 | 1 |
| MB | 4151 | 1 |
| MB21D2 | 151963 | 1 |
| MCEMP1 | 199675 | 1 |
| MCIDAS | 345643 | 1 |
| MEF2C | 4208 | 1 |
| MET | 4233 | 1 |
| METTL21E | 403183 | 1 |
| MFAP4 | 4239 | 1 |
| MGAM | 8972 | 1 |
| MIR133B | 442890 | 1 |
| MIR206 | 406989 | 1 |
| MLIP | 90523 | 1 |
| MME | 4311 | 1 |
| MMP12 | 4321 | 1 |
| MMP13 | 4322 | 1 |
| MMP8 | 4317 | 1 |
| MS4A7 | 58475 | 1 |
| MSTN | 2660 | 1 |
| MTARC1 | 64757 | 1 |
| MTOR | 2475 | 1 |
| MUC1 | 4582 | 1 |
| MUC5B | 727897 | 1 |
| MUCL3 | 135656 | 1 |
| MYBPC1 | 4604 | 1 |
| MYF6 | 4618 | 1 |
| MYH1 | 4619 | 1 |
| MYH11 | 4629 | 1 |
| MYH13 | 8735 | 1 |
| MYH2 | 4620 | 1 |
| MYH4 | 4622 | 1 |
| MYL1 | 4632 | 1 |
| MYL9 | 10398 | 1 |
| MYLK | 4638 | 1 |
| MYLPF | 17907 | 1 |
| MYO18B | 84700 | 1 |
| MYO1C | 4641 | 1 |
| MYOM1 | 8736 | 1 |
| MYOM2 | 9172 | 1 |
| MYOM3 | 127294 | 1 |
| MYOT | 9499 | 1 |
| MYOZ1 | 58529 | 1 |
| MYOZ3 | 91977 | 1 |
| MYPN | 84665 | 1 |
| MZB1 | 51237 | 1 |
| NAALAD2 | 10003 | 1 |
| NDRG1 | 10397 | 1 |
| NEB | 4703 | 1 |
| NEGR1 | 257194 | 1 |
| NEXN | 91624 | 1 |
| NFKBIB | 4793 | 1 |
| NGP | 18054 | 1 |
| NKAIN3 | 286183 | 1 |
| NKX3-1 | 4824 | 1 |
| NMRK2 | 27231 | 1 |
| NOS3 | 4846 | 1 |
| NOX1 | 27035 | 1 |
| NOX4 | 50507 | 1 |
| NPPB | 4879 | 1 |
| NPY | 4852 | 1 |
| NPY1R | 4886 | 1 |
| NR1H2 | 7376 | 1 |
| NR1I2 | 8856 | 1 |
| NR4A1 | 3164 | 1 |
| NR4A3 | 8013 | 1 |
| NRAP | 4892 | 1 |
| NSG2 | 51617 | 1 |
| NUCB2 | 4925 | 1 |
| OBSCN | 84033 | 1 |
| OGG1 | 4968 | 1 |
| OLFML1 | 283298 | 1 |
| OPCML | 4978 | 1 |
| OPTC | 26254 | 1 |
| PAX1 | 5075 | 1 |
| PCDH18 | 54510 | 1 |
| PCK1 | 5105 | 1 |
| PCLO | 27445 | 1 |
| PCOLCE2 | 26577 | 1 |
| PDE6C | 5146 | 1 |
| PDGFRA | 5156 | 1 |
| PDK4 | 5166 | 1 |
| PEG10 | 23089 | 1 |
| PERM1 | 84808 | 1 |
| PGAM2 | 5224 | 1 |
| PGM5 | 5239 | 1 |
| PHACTR1 | 221692 | 1 |
| PHETA2 | 150368 | 1 |
| PHEX | 5251 | 1 |
| PHGR1 | 644844 | 1 |
| PID1 | 55022 | 1 |
| PIEZO2 | 63895 | 1 |
| PIK3C3 | 5289 | 1 |
| PIK3CB | 5291 | 1 |
| PIK3CG | 5294 | 1 |
| PKNOX2 | 63876 | 1 |
| PLAAT1 | 57110 | 1 |
| PLEK | 5341 | 1 |
| PLIN4 | 729359 | 1 |
| PLN | 5350 | 1 |
| PNMA8B | 57469 | 1 |
| POR | 5447 | 1 |
| PPARB | 100136658 | 1 |
| PPBP | 5473 | 1 |
| PPP1R3A | 5506 | 1 |
| PPP1R3C | 5507 | 1 |
| PPP2CA | 5515 | 1 |
| PPP2R2B | 5521 | 1 |
| PRKCG | 5582 | 1 |
| PRKCQ | 5588 | 1 |
| PRKG1 | 5592 | 1 |
| PROK2 | 60675 | 1 |
| PRR32 | 100130613 | 1 |
| PRR33 | 102724536 | 1 |
| PRSS2 | 5645 | 1 |
| PRXL2B | 127281 | 1 |
| PSG20 | 434540 | 1 |
| PSORS1C2 | 170680 | 1 |
| PTEN | 5728 | 1 |
| PTGFR | 5737 | 1 |
| PVALB | 5816 | 1 |
| PXMP2 | 5827 | 1 |
| PYGM | 5837 | 1 |
| RALYL | 138046 | 1 |
| RANBP2 | 5903 | 1 |
| RASD2 | 23551 | 1 |
| RASL12 | 51285 | 1 |
| RB1 | 5925 | 1 |
| RCAN2 | 10231 | 1 |
| RDH10 | 157506 | 1 |
| REEP3 | 221035 | 1 |
| RETNLA | 57262 | 1 |
| RETNLG | 245195 | 1 |
| GC | 28984 | 1 |
| RGS1 | 5996 | 1 |
| RGS13 | 6003 | 1 |
| RGS5 | 8490 | 1 |
| RGS7BP | 401190 | 1 |
| RHEB | 6009 | 1 |
| RIOX1 | 79697 | 1 |
| RNASE2B | 54159 | 1 |
| RNASE6 | 6039 | 1 |
| RNU1-3 | 26869 | 1 |
| RNVU1-18 | 26863 | 1 |
| RNVU1-7 | 26864 | 1 |
| ROBO3 | 64221 | 1 |
| RORC | 6097 | 1 |
| RPL3L | 6123 | 1 |
| RPS6KA1 | 6195 | 1 |
| RPS6KA5 | 9252 | 1 |
| RPTOROS | 319454 | 1 |
| RPUSD2 | 27079 | 1 |
| RSPO1 | 284654 | 1 |
| RTL3 | 203430 | 1 |
| RTN2 | 6253 | 1 |
| RUNX1T1 | 862 | 1 |
| RYR1 | 6261 | 1 |
| SAA3 | 20210 | 1 |
| SBK2 | 646643 | 1 |
| SBK3 | 100130827 | 1 |
| SBSPON | 157869 | 1 |
| SCD | 6319 | 1 |
| SCGB1B3 | 384585 | 1 |
| SCGB2B20 | 494519 | 1 |
| SCGB2B7 | 100043836 | 1 |
| SCGB3A2 | 117156 | 1 |
| SCN2A | 6326 | 1 |
| SCN4A | 6329 | 1 |
| SCN4B | 6330 | 1 |
| SCN7A | 6332 | 1 |
| SCUBE1 | 80274 | 1 |
| SEC31B | 25956 | 1 |
| SELE | 6401 | 1 |
| SELP | 6403 | 1 |
| SERPINB3C | 381286 | 1 |
| SERPINB9B | 20706 | 1 |
| SERPINB9F | 20709 | 1 |
| SFRP2 | 6423 | 1 |
| SFRP4 | 6424 | 1 |
| SFTPA1 | 653509 | 1 |
| SGCA | 6442 | 1 |
| SGCE | 8910 | 1 |
| SGCG | 6445 | 1 |
| SGK1 | 6446 | 1 |
| SH2D1B1 | 26904 | 1 |
| SH2D5 | 400745 | 1 |
| SH2D7 | 646892 | 1 |
| SHE | 126669 | 1 |
| SLC17A8 | 246213 | 1 |
| SLC22A2 | 6582 | 1 |
| SLC25A24 | 29957 | 1 |
| SLC25A34 | 284723 | 1 |
| SLC25A53 | 401612 | 1 |
| SLC26A7 | 115111 | 1 |
| SLC27A1 | 376497 | 1 |
| SLC2A14 | 144195 | 1 |
| SLC2A3 | 6515 | 1 |
| SLC2A4 | 6517 | 1 |
| SLC2A4RG-PS | 329584 | 1 |
| SLC2A6 | 11182 | 1 |
| SLC38A5 | 92745 | 1 |
| SLC4A5 | 57835 | 1 |
| SLC5A7 | 60482 | 1 |
| SLC6A12 | 6539 | 1 |
| SLC6A15 | 55117 | 1 |
| SLC6A17 | 388662 | 1 |
| SLC7A11 | 23657 | 1 |
| SLCO1B1 | 10599 | 1 |
| SLCO1B3 | 28234 | 1 |
| SLCO2B1 | 11309 | 1 |
| SLCO4C1 | 353189 | 1 |
| SLCO5A1 | 81796 | 1 |
| SLIT1 | 6585 | 1 |
| SLN | 6588 | 1 |
| SLTM | 79811 | 1 |
| SMAD9 | 4093 | 1 |
| SMGC | 223809 | 1 |
| SMIM10L2A | 399668 | 1 |
| SMOC1 | 64093 | 1 |
| SMPX | 23676 | 1 |
| SMTNL1 | 219537 | 1 |
| SNCAIP | 9627 | 1 |
| SOD2 | 6648 | 1 |
| SORCS1 | 114815 | 1 |
| SOWAHC | 65124 | 1 |
| SOX10 | 6663 | 1 |
| SOX8 | 30812 | 1 |
| SP7 | 121340 | 1 |
| SPARCL1 | 8404 | 1 |
| SPEG | 10290 | 1 |
| SPINK12 | 78242 | 1 |
| SPOCD1 | 90853 | 1 |
| SPON1 | 10418 | 1 |
| SPRR1B | 6699 | 1 |
| SPRR2B | 6701 | 1 |
| SPRR2D | 6703 | 1 |
| SPRR2E | 6704 | 1 |
| SPRR2F | 6705 | 1 |
| SPRR2G | 6706 | 1 |
| SPRR2H | 20762 | 1 |
| SPRR2I | 20763 | 1 |
| SPRR2J-PS | 20764 | 1 |
| SPRR2K | 20765 | 1 |
| SRL | 6345 | 1 |
| SRPX | 8406 | 1 |
| STAC3 | 246329 | 1 |
| STFA2 | 20862 | 1 |
| STFA2L1 | 268885 | 1 |
| STFA3 | 20863 | 1 |
| STK11 | 6794 | 1 |
| STX1B | 112755 | 1 |
| STYXL2 | 92235 | 1 |
| SULT1C1 | 20888 | 1 |
| SULT1C2 | 6819 | 1 |
| SUSD5 | 26032 | 1 |
| SVEP1 | 79987 | 1 |
| SVOPL | 136306 | 1 |
| SYN1 | 6853 | 1 |
| SYN2 | 6854 | 1 |
| SYNPO2 | 171024 | 1 |
| SYPL2 | 284612 | 1 |
| TARM1 | 441864 | 1 |
| TAS2R108 | 57253 | 1 |
| TBX15 | 6913 | 1 |
| TBX4 | 9496 | 1 |
| TCAP | 8557 | 1 |
| TCEAL7 | 56849 | 1 |
| TDH | 157739 | 1 |
| TDO2 | 6999 | 1 |
| TET1 | 80312 | 1 |
| TFAM | 7019 | 1 |
| TF | 7032 | 1 |
| TG | 7038 | 1 |
| TGM7 | 116179 | 1 |
| THRSP | 7069 | 1 |
| TIMP1 | 7076 | 1 |
| TLE6 | 79816 | 1 |
| TLR11 | 239081 | 1 |
| TM4SF19 | 116211 | 1 |
| TMEM132C | 92293 | 1 |
| TMEM177 | 80775 | 1 |
| TMEM182 | 130827 | 1 |
| TMEM200B | 399474 | 1 |
| TMEM213 | 155006 | 1 |
| TMEM252 | 169693 | 1 |
| TMEM91 | 641649 | 1 |
| TMOD1 | 7111 | 1 |
| TMOD4 | 29765 | 1 |
| TNFAIP6 | 7130 | 1 |
| TNFRSF11B | 4982 | 1 |
| TNFRSF1B | 7133 | 1 |
| TNNC2 | 7125 | 1 |
| TNNI2 | 7136 | 1 |
| TNNT1 | 7138 | 1 |
| TNNT3 | 7140 | 1 |
| TPI1 | 7167 | 1 |
| TRARG1 | 286753 | 1 |
| TRDN | 10345 | 1 |
| TREML1 | 340205 | 1 |
| TRIL | 9865 | 1 |
| TRIM30B | 244183 | 1 |
| TRIM54 | 57159 | 1 |
| TRPC3 | 7222 | 1 |
| TTC41 | 103220 | 1 |
| TTN | 7273 | 1 |
| TUBB3 | 10381 | 1 |
| TXLNB | 167838 | 1 |
| U90926 | 57425 | 1 |
| UBE2U | 148581 | 1 |
| UGT1A1 | 54658 | 1 |
| ULK2 | 9706 | 1 |
| UMPS | 7372 | 1 |
| UNC45B | 146862 | 1 |
| USP13 | 8975 | 1 |
| VAT1L | 57687 | 1 |
| VEGFB | 7423 | 1 |
| VTN | 7448 | 1 |
| VWA2 | 340706 | 1 |
| WFDC13 | 164237 | 1 |
| WFDC15B | 192201 | 1 |
| WFDC17 | 100034251 | 1 |
| WFDC18 | 14038 | 1 |
| WNT2B | 7482 | 1 |
| WSCD2 | 9671 | 1 |
| XCR1 | 2829 | 1 |
| XIRP1 | 165904 | 1 |
| XIRP2 | 129446 | 1 |
| XRCC1 | 7515 | 1 |
| YIPF7 | 285525 | 1 |
| ZC3H11A | 9877 | 1 |
| ZCCHC18 | 644353 | 1 |
| ZCWPW1 | 55063 | 1 |
| ZDBF2 | 57683 | 1 |
| ZFP354C | 30944 | 1 |
| ZFP36 | 7538 | 1 |
| ZFP385C | 278304 | 1 |
| ZFP454 | 237758 | 1 |
| ZFP521 | 225207 | 1 |
| ZFP641 | 239652 | 1 |
| ZFP951 | 626391 | 1 |
| ZKSCAN4 | 387032 | 1 |
| ZNF117 | 51351 | 1 |
| ZNF681 | 148213 | 1 |
| ZPBP | 11055 | 1 |

| **Biological Process P-Value Count** |
| --- |

**Table S3.** Biological processes.

| positive regulation of gene expression | 0.049628203 | 14 |
| --- | --- | --- |
| positive regulation of transcription, DNA-templated | 0.049628203 | 10 |
| positive regulation of transcription from RNA polymerase II promoter | 0.049628203 | 10 |
| inflammatory response | 0.047858886 | 9 |
| response to xenobiotic stimulus | 0.048501299 | 9 |
| positive regulation of apoptotic process | 0.04398089 | 8 |
| negative regulation of apoptotic process | 0.04398089 | 8 |
| positive regulation of cell proliferation | 0.04398089 | 8 |
| cellular response to hypoxia | 0.046243656 | 8 |
| negative regulation of gene expression | 0.046243656 | 8 |
| positive regulation of smooth muscle cell proliferation | 0.04398089 | 7 |
| signal transduction | 0.04398089 | 7 |
| positive regulation of cell migration | 0.038301487 | 6 |
| negative regulation of cell proliferation | 0.038301487 | 6 |
| positive regulation of MAPK cascade | 0.039439943 | 6 |
| positive regulation of protein kinase B signaling | 0.039439943 | 6 |
| negative regulation of neuron apoptotic process | 0.04057711 | 6 |
| regulation of blood pressure | 0.041712989 | 6 |
| positive regulation of sequence-specific DNA binding transcription factor activity | 0.041712989 | 6 |
| aging | 0.041712989 | 6 |
| positive regulation of mitotic nuclear division | 0.042847582 | 6 |
| liver regeneration | 0.042847582 | 6 |
| positive regulation of angiogenesis | 0.031443599 | 5 |
| angiogenesis | 0.031443599 | 5 |
| negative regulation of transcription from RNA polymerase II promoter | 0.031443599 | 5 |
| positive regulation of peptidyl-tyrosine phosphorylation | 0.032589819 | 5 |
| response to lipopolysaccharide | 0.032589819 | 5 |
| protein kinase B signaling | 0.033734742 | 5 |
| positive regulation of peptidyl-serine phosphorylation | 0.033734742 | 5 |
| response to glucocorticoid | 0.034878369 | 5 |
| positive regulation of cytokine production | 0.034878369 | 5 |
| positive regulation of MAP kinase activity | 0.034878369 | 5 |
| positive regulation of pri-miRNA transcription from RNA polymerase II promoter | 0.036020701 | 5 |
| positive regulation of vascular endothelial growth factor production | 0.038301487 | 5 |
| positive regulation of protein phosphorylation | 0.022226953 | 4 |
| positive regulation of ERK1 and ERK2 cascade | 0.022226953 | 4 |
| regulation of gene expression | 0.022226953 | 4 |
| protein phosphorylation | 0.022226953 | 4 |
| response to oxidative stress | 0.023383604 | 4 |
| defense response to Gram-positive bacterium | 0.023383604 | 4 |
| positive regulation of NF-kappaB transcription factor activity | 0.023383604 | 4 |
| response to hypoxia | 0.023383604 | 4 |
| cellular response to lipopolysaccharide | 0.023383604 | 4 |
| glucose homeostasis | 0.024157093 | 4 |
| response to ethanol | 0.024538947 | 4 |
| positive regulation of phosphatidylinositol 3-kinase signaling | 0.025692983 | 4 |
| response to estradiol | 0.025692983 | 4 |
| positive regulation of interleukin-6 production | 0.025692983 | 4 |
| epithelial to mesenchymal transition | 0.026845712 | 4 |
| positive regulation of vascular smooth muscle cell proliferation | 0.026845712 | 4 |
| positive regulation of epithelial to mesenchymal transition | 0.026845712 | 4 |
| cellular response to hydrogen peroxide | 0.026845712 | 4 |
| positive regulation of nitric oxide biosynthetic process | 0.027997137 | 4 |
| positive regulation of blood vessel endothelial cell migration | 0.027997137 | 4 |
| response to activity | 0.027997137 | 4 |
| response to heat | 0.027997137 | 4 |
| positive regulation of chemokine production | 0.029147259 | 4 |
| cellular response to beta-amyloid | 0.029147259 | 4 |
| embryo implantation | 0.029480864 | 4 |
| positive regulation of glycolytic process | 0.030296079 | 4 |
| negative regulation of extrinsic apoptotic signaling pathway via death domain receptors | 0.030296079 | 4 |
| lipopolysaccharide-mediated signaling pathway | 0.030296079 | 4 |
| negative regulation of lipid storage | 0.030851428 | 4 |
| vascular endothelial growth factor production | 0.031443599 | 4 |
| cell-cell signaling | 0.003541364 | 3 |
| cellular response to DNA damage stimulus | 0.003541364 | 3 |
| peptidyl-serine phosphorylation | 0.003798792 | 3 |
| positive regulation of I-kappaB kinase/NF-kappaB signaling | 0.003798792 | 3 |
| MAPK cascade | 0.003847642 | 3 |
| negative regulation of inflammatory response | 0.00399167 | 3 |
| positive regulation of inflammatory response | 0.004719151 | 3 |
| negative regulation of cell growth | 0.004719151 | 3 |
| wound healing | 0.00524243 | 3 |
| cellular response to virus | 0.005579839 | 3 |
| cellular response to retinoic acid | 0.005895605 | 3 |
| positive regulation of endothelial cell proliferation | 0.005895605 | 3 |
| cellular response to mechanical stimulus | 0.005895605 | 3 |
| rhythmic process | 0.005895605 | 3 |
| positive regulation of cell growth | 0.005895605 | 3 |
| peptidyl-threonine phosphorylation | 0.005927001 | 3 |
| response to ischemia | 0.00707073 | 3 |
| positive regulation of tyrosine phosphorylation of STAT protein | 0.00707073 | 3 |
| cellular response to xenobiotic stimulus | 0.00707073 | 3 |
| positive regulation of NIK/NF-kappaB signaling | 0.00707073 | 3 |
| cellular iron ion homeostasis | 0.007940484 | 3 |
| response to nutrient | 0.008244526 | 3 |
| negative regulation of autophagy | 0.008244526 | 3 |
| response to estrogen | 0.008244526 | 3 |
| humoral immune response | 0.008244526 | 3 |
| I-kappaB kinase/NF-kappaB signaling | 0.008244526 | 3 |
| regulation of circadian rhythm | 0.008244526 | 3 |
| positive regulation of interleukin-8 production | 0.008244526 | 3 |
| ERK1 and ERK2 cascade | 0.009416995 | 3 |
| positive regulation of protein complex assembly | 0.009416995 | 3 |
| negative regulation of fat cell differentiation | 0.009416995 | 3 |
| response to nutrient levels | 0.00962536 | 3 |
| cellular response to organic cyclic compound | 0.010588137 | 3 |
| positive regulation of protein secretion | 0.010588137 | 3 |
| positive regulation of phagocytosis | 0.010588137 | 3 |
| intrinsic apoptotic signaling pathway in response to DNA damage | 0.010588137 | 3 |
| extrinsic apoptotic signaling pathway | 0.010588137 | 3 |
| regulation of insulin secretion | 0.011757955 | 3 |
| positive regulation of JAK-STAT cascade | 0.011757955 | 3 |
| vasodilation | 0.012926451 | 3 |
| negative regulation of vascular smooth muscle cell proliferation | 0.012926451 | 3 |
| response to hydrogen peroxide | 0.013257064 | 3 |
| negative regulation of extrinsic apoptotic signaling pathway in absence of ligand | 0.014093624 | 3 |
| negative regulation of blood vessel endothelial cell migration | 0.014093624 | 3 |
| response to nicotine | 0.014093624 | 3 |
| acute-phase response | 0.014093624 | 3 |
| cell redox homeostasis | 0.014093624 | 3 |
| negative regulation of endothelial cell apoptotic process | 0.014640075 | 3 |
| positive regulation of glucose import | 0.014971221 | 3 |
| positive regulation of DNA binding | 0.015259477 | 3 |
| extrinsic apoptotic signaling pathway in absence of ligand | 0.015259477 | 3 |
| response to amino acid | 0.016424012 | 3 |
| activation of protein kinase B activity | 0.016424012 | 3 |
| positive regulation of cytokine production involved in inflammatory response | 0.016424012 | 3 |
| negative regulation of smooth muscle cell proliferation | 0.016424012 | 3 |
| positive regulation of nitric-oxide synthase activity | 0.017587229 | 3 |
| muscle cell cellular homeostasis | 0.017587229 | 3 |
| negative regulation of pri-miRNA transcription from RNA polymerase II promoter | 0.017587229 | 3 |
| negative regulation of neurogenesis | 0.01874913 | 3 |
| cellular response to organic substance | 0.01874913 | 3 |
| positive regulation of glial cell proliferation | 0.019743545 | 3 |
| response to iron ion | 0.019909717 | 3 |
| negative regulation of lipid catabolic process | 0.019909717 | 3 |
| response to vitamin D | 0.019909717 | 3 |
| positive regulation of superoxide anion generation | 0.019909717 | 3 |
| positive regulation of fever generation | 0.021068991 | 3 |
| positive regulation of receptor activity | 0.021068991 | 3 |
| removal of superoxide radicals | 0.021068991 | 3 |
| positive regulation of heterotypic cell-cell adhesion | 0.021068991 | 3 |
| lactation | 2.62E-15 | 2 |
| positive regulation of JUN kinase activity | 5.80E-11 | 2 |
| positive regulation of protein import into nucleus | 1.40E-10 | 2 |
| ovarian follicle development | 2.02E-10 | 2 |
| positive regulation of protein localization to nucleus | 5.33E-10 | 2 |
| extrinsic apoptotic signaling pathway via death domain receptors | 6.20E-10 | 2 |
| phosphatidylinositol 3-kinase signaling | 2.17E-08 | 2 |
| T cell homeostasis | 3.96E-08 | 2 |
| neuronal action potential | 4.57E-08 | 2 |
| reactive oxygen species metabolic process | 5.03E-08 | 2 |
| placenta development | 5.82E-08 | 2 |
| regulation of angiogenesis | 7.13E-08 | 2 |
| regulation of I-kappaB kinase/NF-kappaB signaling | 8.47E-08 | 2 |
| response to amphetamine | 1.21E-07 | 2 |
| cellular response to cadmium ion | 2.32E-07 | 2 |
| aortic valve morphogenesis | 4.18E-07 | 2 |
| positive regulation of cell migration involved in sprouting angiogenesis | 6.92E-07 | 2 |
| response to testosterone | 6.92E-07 | 2 |
| negative regulation of apoptotic signaling pathway | 1.15E-06 | 2 |
| regulation of mitochondrial membrane potential | 1.29E-06 | 2 |
| maintenance of permeability of blood-brain barrier | 1.50E-06 | 2 |
| positive regulation of macroautophagy | 1.89E-06 | 2 |
| homeostasis of number of cells within a tissue | 2.08E-06 | 2 |
| positive regulation of peptidyl-threonine phosphorylation | 2.15E-06 | 2 |
| positive regulation of catalytic activity | 2.16E-06 | 2 |
| negative regulation of mitotic cell cycle | 2.32E-06 | 2 |
| negative regulation of myoblast differentiation | 2.87E-06 | 2 |
| positive regulation of oligodendrocyte differentiation | 4.04E-06 | 2 |
| positive regulation of histone acetylation | 4.58E-06 | 2 |
| positive regulation of SMAD protein import into nucleus | 4.63E-06 | 2 |
| positive regulation of immunoglobulin production | 5.76E-06 | 2 |
| response to immobilization stress | 1.29E-05 | 2 |
| regulation of synapse organization | 1.33E-05 | 2 |
| regulation of multicellular organism growth | 1.61E-05 | 2 |
| response to radiation | 1.98E-05 | 2 |
| decidualization | 1.98E-05 | 2 |
| positive regulation of interleukin-17 production | 2.12E-05 | 2 |
| cell aging | 2.26E-05 | 2 |
| superoxide anion generation | 2.56E-05 | 2 |
| positive regulation of protein metabolic process | 2.61E-05 | 2 |
| negative regulation of protein localization to nucleus | 2.73E-05 | 2 |
| cellular response to low-density lipoprotein particle stimulus | 2.73E-05 | 2 |
| negative regulation of reactive oxygen species metabolic process | 3.25E-05 | 2 |
| positive regulation of smooth muscle cell migration | 3.63E-05 | 2 |
| nitric oxide biosynthetic process | 3.63E-05 | 2 |
| positive regulation of cardiac muscle hypertrophy | 3.98E-05 | 2 |
| positive regulation of lipid biosynthetic process | 7.18E-05 | 2 |
| TOR signaling | 8.75E-05 | 2 |
| positive regulation of calcineurin-NFAT signaling cascade | 1.08E-04 | 2 |
| negative regulation of ossification | 1.21E-04 | 2 |
| positive regulation of protein export from nucleus | 1.39E-04 | 2 |
| cellular response to ATP | 1.74E-04 | 2 |
| regulation of cellular response to heat | 1.80E-04 | 2 |
| prostaglandin biosynthetic process | 2.02E-04 | 2 |
| negative regulation of oxidative stress-induced intrinsic apoptotic signaling pathway | 2.09E-04 | 2 |
| vasoconstriction | 2.23E-04 | 2 |
| regulation of sodium ion transport | 2.26E-04 | 2 |
| epithelial cell apoptotic process | 2.50E-04 | 2 |
| regulation of protein secretion | 2.51E-04 | 2 |
| positive regulation of vascular endothelial growth factor receptor signaling pathway | 3.04E-04 | 2 |
| positive regulation of cellular protein metabolic process | 3.10E-04 | 2 |
| positive regulation of membrane protein ectodomain proteolysis | 3.63E-04 | 2 |
| ectopic germ cell programmed cell death | 3.63E-04 | 2 |
| mammary gland alveolus development | 3.94E-04 | 2 |
| astrocyte activation | 3.98E-04 | 2 |
| negative regulation of epithelial cell apoptotic process | 4.17E-04 | 2 |
| negative regulation of macroautophagy | 4.61E-04 | 2 |
| positive regulation of smooth muscle contraction | 4.61E-04 | 2 |
| positive regulation of brown fat cell differentiation | 4.96E-04 | 2 |
| negative regulation of calcineurin-NFAT signaling cascade | 5.69E-04 | 2 |
| negative regulation of collagen biosynthetic process | 6.08E-04 | 2 |
| positive regulation of glycogen biosynthetic process | 6.48E-04 | 2 |
| cellular response to fluid shear stress | 6.90E-04 | 2 |
| positive regulation of transcription regulatory region DNA binding | 6.90E-04 | 2 |
| positive regulation of leukocyte adhesion to vascular endothelial cell | 7.76E-04 | 2 |
| negative regulation of oxidative stress-induced neuron intrinsic apoptotic signaling pathway | 8.68E-04 | 2 |
| negative regulation of macrophage derived foam cell differentiation | 8.68E-04 | 2 |
| digestive tract morphogenesis | 8.68E-04 | 2 |
| regulation of osteoclast differentiation | 9.19E-04 | 2 |
| positive regulation of chemokine (C-X-C motif) ligand 2 production | 0.001013974 | 2 |
| positive regulation of prostaglandin secretion | 0.001065207 | 2 |
| response to lithium ion | 0.001117665 | 2 |
| negative regulation of calcium ion transport | 0.001145593 | 2 |
| positive regulation of production of miRNAs involved in gene silencing by miRNA | 0.001226241 | 2 |
| negative regulation of production of miRNAs involved in gene silencing by miRNA | 0.001282354 | 2 |
| negative regulation of developmental process | 0.00133968 | 2 |
| negative regulation of smooth muscle cell apoptotic process | 0.00133968 | 2 |
| endothelial cell apoptotic process | 0.00133968 | 2 |
| negative regulation of biomineral tissue development | 0.001398217 | 2 |
| response to carbohydrate | 0.001518912 | 2 |
| positive regulation of cell growth involved in cardiac muscle cell development | 0.001580797 | 2 |
| cellular response to nutrient | 0.001581065 | 2 |
| regulation of establishment of endothelial barrier | 0.001581065 | 2 |
| negative regulation of acute inflammatory response | 0.001708967 | 2 |
| negative regulation of receptor activity | 0.001708967 | 2 |
| macrophage derived foam cell differentiation | 0.001774711 | 2 |
| regulation of transmembrane transporter activity | 0.001841646 | 2 |
| negative regulation of cytokine production involved in immune response | 0.001909771 | 2 |
| inflammatory response to wounding | 0.002305014 | 2 |
| response to salt | 0.002343344 | 2 |
| response to leptin | 0.002362244 | 2 |
| heart valve morphogenesis | 0.002362244 | 2 |
| positive regulation of prostaglandin biosynthetic process | 0.002362244 | 2 |
| positive regulation of transcription from RNA polymerase II promoter in response to hypoxia | 0.002419719 | 2 |
| positive regulation of STAT cascade | 0.002422684 | 2 |
| regulation of transcription from RNA polymerase II promoter in response to oxidative stress | 0.002497262 | 2 |
| regulation of systemic arterial blood pressure by endothelin | 0.002497262 | 2 |
| connective tissue replacement involved in inflammatory response wound healing | 0.00257597 | 2 |
| positive regulation of odontogenesis | 0.00257597 | 2 |
| negative regulation of gene silencing by miRNA | 0.002655841 | 2 |
| response to fructose | 0.002655841 | 2 |
| positive regulation of mononuclear cell migration | 0.002655841 | 2 |
| sequestering of triglyceride | 0.002736872 | 2 |
| response to macrophage colony-stimulating factor | 0.002902404 | 2 |
| response to carbon monoxide | 0.003159339 | 2 |
| positive regulation of chemokine-mediated signaling pathway | 0.003247276 | 2 |
| chronic inflammatory response to antigenic stimulus | 0.003247276 | 2 |
| positive regulation of calcidiol 1-monooxygenase activity | 0.003247276 | 2 |
| smooth muscle hyperplasia | 0.00343414 | 2 |

**Table S4.** Cellular compartments.

| **Cellular Compartment** | **P-value** | **Count** |
| --- | --- | --- |

| extracellular space | 2.66E-08 | 14 |
| --- | --- | --- |
| cytoplasm | 0.012399309 | 13 |
| extracellular region | 8.24E-06 | 12 |
| cytosol | 0.027938757 | 12 |
| nucleus | 0.049284342 | 12 |
| macromolecular complex | 0.006993178 | 5 |
| endoplasmic reticulum lumen | 0.004785989 | 4 |
| RNA polymerase II transcription factor complex | 0.008325217 | 3 |
| mitochondrial outer membrane | 0.023828858 | 3 |
| lysosome | 0.0475006 | 3 |
| neuronal cell body | 0.008500792 | 4 |
| platelet alpha granule lumen | 0.002582639 | 3 |
| caveola | 0.003131259 | 3 |

**Table S5.** Molecular functions.

| **Molecular Function** | **P-value** | **Count** |
| --- | --- | --- |

| protein binding | 8.95E-05 | 24 |
| --- | --- | --- |
| identical protein binding | 1.10E-04 | 10 |
| cytokine activity | 8.68E-08 | 7 |
| enzyme binding | 8.65E-05 | 6 |
| growth factor activity | 4.50E-05 | 5 |
| protease binding | 3.34E-04 | 4 |
| integrin binding | 9.20E-04 | 4 |
| ubiquitin protein ligase binding | 0.006273293 | 4 |
| sequence-specific DNA binding | 0.008120135 | 4 |
| RNA polymerase II sequence-specific DNA binding transcription factor binding | 0.021334467 | 3 |
| heme binding | 0.014987955 | 3 |
| hormone activity | 0.008195083 | 3 |
| transcription regulatory region sequence-specific DNA binding | 0.033591426 | 3 |
| protein domain specific binding | 0.037537498 | 3 |
| superoxide dismutase activity | 0.006046249 | 2 |
| insulin-like growth factor receptor binding | 0.019225151 | 2 |
| insulin receptor binding | 0.027524522 | 2 |
| transcription cofactor binding | 0.04392218 | 2 |

**Table S6.** Reactome pathways.

| **Reactome Pathway** | **P-value** | **Count** |
| --- | --- | --- |

|  | |  |  |
| --- | --- | --- | --- |
| Immune System | | 8.88E-06 | 15 |
| Signal Transduction | 1.40E-04 | | 15 |
| Signaling by Interleukins | | 6.95E-10 | 12 |
| Cytokine Signaling in Immune system | | 1.42E-07 | 12 |
| Interleukin-4 and Interleukin-13 signaling | | 5.61E-15 | 11 |
| Cellular responses to stress | | 1.72E-05 | 10 |
| Cellular responses to stimuli | | 1.98E-05 | 10 |
| Disease | | 0.006123377 | 10 |
| Generic Transcription Pathway | | 0.01143046 | 8 |
| RNA Polymerase II Transcription | | 0.018716371 | 8 |
| Gene expression (Transcription) | | 0.033316087 | 8 |
| Cellular response to chemical stress | | 3.96E-06 | 7 |
| Signaling by Receptor Tyrosine Kinases | | 5.81E-04 | 7 |
| Infectious disease | | 0.011996666 | 7 |
| Interleukin-10 signaling | | 3.62E-08 | 6 |
| Nuclear events mediated by NFE2L2 | | 1.42E-06 | 6 |
| KEAP1-NFE2L2 pathway | | 5.17E-06 | 6 |
| PIP3 activates AKT signaling | | 0.002110646 | 5 |
| Intracellular signaling by second messengers | | 0.003575533 | 5 |
| Hemostasis | | 0.037748544 | 5 |
| Extra-nuclear estrogen signaling | | 5.27E-04 | 4 |
| Platelet degranulation | | 0.002345104 | 4 |
| Response to elevated platelet cytosolic Ca2+ | | 0.002612978 | 4 |
| Parasitic Infection Pathways | | 0.004617849 | 4 |
| Leishmania infection | | 0.004617849 | 4 |
| ESR-mediated signaling | | 0.010637974 | 4 |
| Signaling by Nuclear Receptors | | 0.023293219 | 4 |
| NFE2L2 regulating tumorigenic genes | | 2.27E-04 | 3 |
| NFE2L2 regulating anti-oxidant/detoxification enzymes | | 6.99E-04 | 3 |
| Regulation of HMOX1 expression and activity | | 0.010404325 | 2 |
| Estrogen-dependent nuclear events downstream of ESR-membrane signaling | | 0.001120611 | 3 |
| Signaling by Non-Receptor Tyrosine Kinases | | 0.005593186 | 3 |
| Signaling by PTK6 | | 0.005593186 | 3 |
| Transcriptional regulation of white adipocyte differentiation | | 0.013118024 | 3 |
| Transcriptional regulation by RUNX3 | | 0.016901608 | 3 |
| PI3K/AKT Signaling in Cancer | | 0.019652729 | 3 |
| Regulation of Insulin-like Growth Factor (IGF) transport and uptake by Insulin-like Growth Factor Binding Proteins (IGFBPs) | | 0.027694977 | 3 |
| Platelet activation, signaling and aggregation | | 0.016766658 | 4 |
| RUNX3 regulates p14-ARF | | 0.018653152 | 2 |
| CD163 mediating an anti-inflammatory response | | 0.018653152 | 2 |
| Synthesis, secretion, and deacylation of Ghrelin | | 0.038988471 | 2 |
| Inflammasomes | | 0.043006877 | 2 |

**Table S7.** Top 10 TFs regulating the hub genes.

| **Rank** | **Transcription Factor** | **Overlapping Genes** |
| --- | --- | --- |
| 1 | CTCF | SPP1, ICAM1, BCL2, NFE2L2, IL6, TGFB1 |
| 2 | TRIM28 | NFE2L2, NOS3 |
| 3 | RELA | NFE2L2, ICAM1, GSK3B |
| 4 | TRIM28 | HIF1A, GSK3B |
| 5 | RAD21 | SPP1, NFE2L2, ICAM1, IL6 |
| 6 | PBX3 | NFE2L2, GSK3B, HIF1A, MTOR |
| 7 | E2F1 | NFE2L2, ICAM1, HIF1A |
| 8 | CBX3 | NOS3 |
| 9 | NFE2L2 | PTGS2, IGF1, HMOX1 |
| 10 | SMC3 | NFE2L2, IL6, TGFB1 |


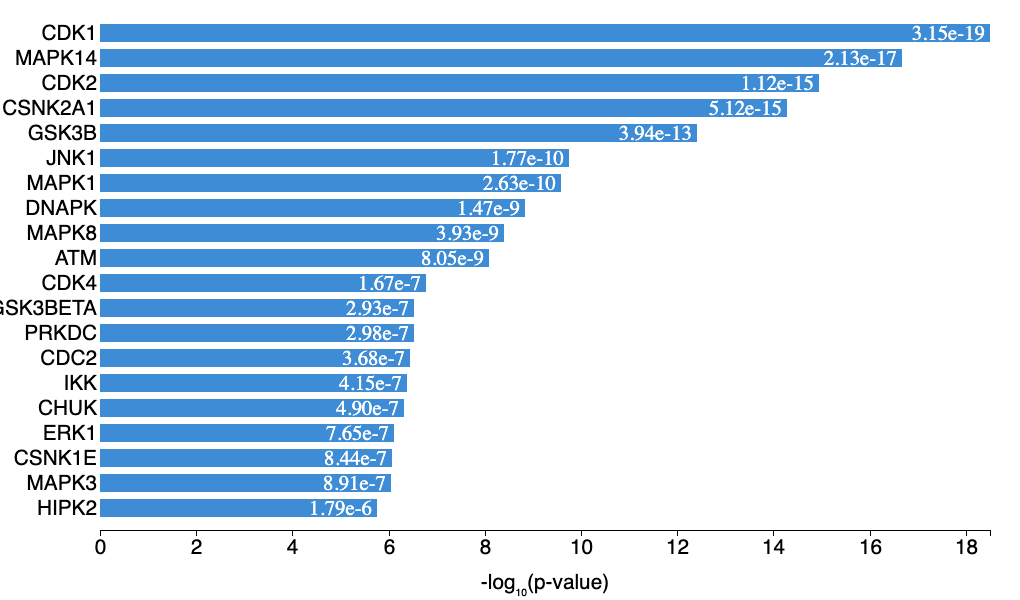


**Figure S1.** Top PKs regulating the hub genes.

**Table S8.** Top 10 miRNAs regulating hub genes.

| **Id** | **miRNA** | **Targets** |
| --- | --- | --- |
| MIMAT0000080 | miR-24-3p | IL1B, TNF, IGF1, NOS3, HMOX1, TGFB1 |
| MIMAT0000765 | miR-335-5p | ICAM1, NOS3, HMOX1, PTGS2, SPP1, IL6 |
| MIMAT0000069 | miR-16-5p | GSK3B, MTOR, HMOX1, PTGS2, BCL2 |
| MIMAT0000070 | miR-17-5p | ICAM1, TGFB1, BCL2, TNF, HIF1A |
| MIMAT0000076 | miR-21-5p | IL1B, HIF1A, ICAM1, TGFB1, BCL2 |
| MIMAT0000083 | miR-26b-5p | NFE2L2, IGF1, HMOX1, PTGS2, GSK3B |
| MIMAT0000084 | miR-27a-3p | HIF1A, IGF1, NFE2L2, PPARG, GSK3B |
| MIMAT0000096 | miR-98-5p | IFG1, BCL2, IL10, IL6, ICAM1 |
| MIMAT0000103 | miR-106a-5p | IL1B, IL6, IL10, HIFA1A, TGFB1 |
| MIMAT0000449 | miR-146a-5p | IL6, ICAM1, SPP1, PTGS2, TGFB1 |
