# Supplementary material for: The ameliorative effect of pioglitazone against colistin-induced nephrotoxicity is mediated by inhibition of NF-κB and restoration of Nrf2 signaling: An integrative bioinformatics prediction-guided in vitro study
Source: PLoS One. 2024 Dec 2;19(12):e0314092. doi: 10.1371/journal.pone.0314092 (PMC11611169; doi:10.1371/journal.pone.0314092)

Experiment 1

	1	2	3	4	5	6
A	0.049	0.039	0.04	0.039	0.04	0.039
B	0.042	0.041	0.114	0.043	0.041	0.041
C	0.042	0.623	0.704	0.6	0.57	0.747
D	0.048	0.573	0.705	0.738	0.714	0.569
E	0.04	0.537	0.603	0.548	0.67	0.598
F	0.04	0.04	0.048	0.042	0.04	0.041
G	0.041	0.043	0.053	0.041	0.04	0.041
H	0.041	0.042	0.048	0.042	0.045	0.043

Experiment 2

	1	2	3	4	5	6
A	0.04	0.04	0.04	0.039	0.039	0.039
B	0.04	0.042	0.041	0.04	0.041	0.04
C	0.041	0.432	0.584	0.54	0.511	0.634
D	0.041	0.445	0.59	0.497	0.449	0.523
E	0.042	0.363	0.535	0.512	0.52	0.535
F	0.04	0.041	0.04	0.048	0.045	0.04
G	0.05	0.041	0.039	0.04	0.041	0.04
H	0.042	0.042	0.045	0.043	0.042	0.041

Experiment 3

	1	2	3	4	5	6
A	0.039	0.041	0.041	0.038	0.037	0.041
B	0.04	0.041	0.042	0.041	0.042	0.041
C	0.042	0.399	0.592	0.52	0.535	0.518
D	0.037	0.421	0.543	0.56	0.441	0.432
E	0.039	0.398	0.562	0.512	0.462	0.571
F	0.041	0.042	0.041	0.049	0.044	0.041
G	0.042	0.043	0.039	0.04	0.041	0.041
H	0.041	0.043	0.046	0.042	0.041	0.042

7	8	9	10	11	12
0.039	0.039	0.04	0.04	0.039	0.042
0.04	0.04	0.04	0.041	0.04	0.04
0.605	0.429	0.334	0.165	0.093	0.04
0.643	0.439	0.265	0.122	0.083	0.04
0.655	0.39	0.274	0.114	0.069	0.041
0.043	0.04	0.062	0.052	0.074	0.04
0.04	0.043	0.061	0.05	0.059	0.04
0.042	0.044	0.042	0.042	0.041	0.041

7	8	9	10	11	12
0.04	0.04	0.04	0.039	0.04	0.039
0.044	0.04	0.04	0.04	0.043	0.04
0.516	0.371	0.314	0.13	0.122	0.04
0.508	0.372	0.278	0.12	0.108	0.041
0.435	0.296	0.225	0.094	0.087	0.04
0.04	0.039	0.043	0.042	0.041	0.039
0.04	0.04	0.055	0.051	0.048	0.039
0.042	0.041	0.043	0.048	0.042	0.041

7	8	9	10	11	12
0.041	0.042	0.04	0.039	0.041	0.039
0.046	0.046	0.046	0.041	0.042	0.041
0.519	0.362	0.201	0.081	0.041	0.041
0.511	0.379	0.203	0.091	0.059	0.041
0.421	0.333	0.2	0.099	0.058	0.041
0.042	0.038	0.044	0.041	0.042	0.038
0.042	0.041	0.052	0.049	0.042	0.042
0.043	0.042	0.042	0.041	0.041	0.049

Blank	Colistin	SFM	500 uM	750 uM	1000 uM	1500 uM
0.041		0.704	0.605	0.429	0.334	0.165
0.043		0.705	0.643	0.439	0.265	0.122
0.04		0.623	0.655	0.39	0.274	0.114
0.062						
0.052		0.652	0.553	0.377	0.282	0.113
0.074		0.653	0.591	0.387	0.213	0.07
0.052		0.571	0.603	0.338	0.222	0.062
		0.62533333	0.58233333	0.36733333	0.239	0.08166667
		104.264448	88.432883	60.2878786	45.0959729	18.0703721
		104.424363	94.5096453	61.8870266	34.0618518	11.1940358
		91.3113493	96.4286228	54.0512015	35.501085	9.91471744
		100.000053	93.123717	58.7420356	38.2196366	13.0597085

Blank	Colistin	SFM	500 uM	750 uM	1000 uM	1500 uM
0.04		0.584	0.516	0.371	0.314	0.13
0.04		0.59	0.508	0.372	0.278	0.12
0.039		0.535	0.435	0.296	0.225	0.094
0.043						
0.042		0.54316667	0.47516667	0.33016667	0.27316667	0.08916667
0.041		0.54916667	0.46716667	0.33116667	0.23716667	0.07916667
0.04083333		0.49416667	0.39416667	0.25516667	0.18416667	0.05316667
		0.52883334	0.4455	0.3055	0.2315	0.07383334
		102.710368	89.8518747	62.4330285	51.6545855	16.8610152
		103.844941	88.3391108	62.622124	44.8471479	14.9700603
		93.444689	74.5351399	48.2508667	34.8250869	10.0535776
		99.9999994	84.2420418	57.7686731	43.7756068	13.961551

Blank	Colistin	SFM	500 uM	750 uM	1000 uM	1500 uM
0.041		0.592	0.519	0.362	0.201	0.081
0.042		0.543	0.511	0.379	0.203	0.091
0.038		0.562	0.421	0.333	0.2	0.099
0.044		0.56566667	0.48366667	0.358	0.20133333	0.09033333
0.041		104.655273	91.7501468	63.9952854	35.5332938	14.3193871
0.042		95.9929281	90.3358863	67.0005889	35.886859	16.0872126
0.04133333		99.3517967	74.4254562	58.8685913	35.3565113	17.5014731
		99.9999994	85.5038298	63.2881552	35.5922214	15.9693576

2000 uM
0.093
0.083
0.069

0.041
0.031
0.017
0.02966667
6.55650669
4.95735872
2.71855156
4.74413899

2000 uM
0.122
0.108
0.087

0.08116667
0.06716667
0.04616667
0.06483334
15.3482513
12.7009144
8.72990912
12.2596916

2000 uM
0.041
0.059
0.058

0.05266667
7.24808481
10.4301708
10.2533883
9.31054797

Experiment 1

	1	2	3	4	5	6
A	0.042	0.039	0.039	0.038	0.04	0.043
B	0.04	0.041	0.043	0.042	0.041	0.041
C	0.04	0.34	0.311	0.116	0.162	0.237
D	0.04	0.378	0.303	0.115	0.127	0.224
E	0.04	0.389	0.337	0.113	0.119	0.19
F	0.04	0.035	0.044	0.036	0.041	0.046
G	0.039	0.04	0.038	0.042	0.041	0.037
H	0.039	0.042	0.037	0.043	0.042	0.045

Experiment 2

	1	2	3	4	5	6
A	0.04	0.04	0.039	0.04	0.04	0.038
B	0.037	0.044	0.039	0.041	0.038	0.043
C	0.04	0.311	0.287	0.092	0.107	0.123
D	0.044	0.262	0.24	0.1	0.077	0.153
E	0.04	0.249	0.248	0.1	0.111	0.127
F	0.039	0.04	0.038	0.041	0.041	0.056
G	0.042	0.042	0.041	0.046	0.04	0.04
H	0.041	0.049	0.042	0.044	0.04	0.042

Experiment 3

	1	2	3	4	5	6
A	0.039	0.043	0.039	0.039	0.042	0.044
B	0.04	0.045	0.041	0.042	0.041	0.042
C	0.039	0.333	0.286	0.113	0.131	0.227
D	0.04	0.352	0.282	0.109	0.116	0.21
E	0.039	0.388	0.309	0.112	0.119	0.195
F	0.04	0.039	0.04	0.041	0.041	0.049
G	0.039	0.039	0.039	0.041	0.041	0.041
H	0.041	0.041	0.042	0.041	0.041	0.041

7	8	9	10	11	12
0.041	0.042	0.043	0.039	0.04	0.04
0.041	0.041	0.04	0.041	0.041	0.041
0.267	0.219	0.226	0.25	0.238	0.041
0.223	0.197	0.24	0.276	0.246	0.04
0.214	0.137	0.232	0.283	0.234	0.037
0.055	0.057	0.084	0.08	0.101	0.042
0.054	0.076	0.039	0.043	0.044	0.041
0.043	0.046	0.043	0.041	0.043	0.042

7	8	9	10	11	12
0.04	0.039	0.038	0.04	0.048	0.04
0.042	0.04	0.039	0.041	0.043	0.043
0.165	0.181	0.211	0.241	0.23	0.04
0.157	0.19	0.161	0.178	0.184	0.04
0.131	0.215	0.159	0.212	0.189	0.039
0.063	0.064	0.077	0.07	0.068	0.042
0.037	0.042	0.042	0.041	0.038	0.04
0.043	0.043	0.041	0.038	0.039	0.038

7	8	9	10	11	12
0.04	0.042	0.041	0.04	0.041	0.04
0.04	0.04	0.041	0.04	0.041	0.04
0.263	0.216	0.229	0.226	0.243	0.04
0.221	0.185	0.305	0.218	0.241	0.04
0.225	0.134	0.186	0.206	0.204	0.039
0.055	0.059	0.066	0.074	0.066	0.043
0.045	0.07	0.04	0.039	0.04	0.04
0.042	0.044	0.041	0.041	0.043	0.042

Blank
0.046
0.055
0.057
0.084
0.08
0.101
0.0705

SFM	Colistin 1200	Pio 100	Pio 150	Pio 200
0.34	0.116	0.237	0.219	0.25
0.378	0.115	0.224	0.197	0.276
0.389	0.113	0.19	0.137	0.283
0.311	0.162	0.267	0.226	0.238
0.303	0.127	0.223	0.24	0.246
0.337	0.119	0.214	0.232	0.234

0.2695	0.0455	0.1665	0.1485	0.1795
0.3075	0.0445	0.1535	0.1265	0.2055
0.3185	0.0425	0.1195	0.0665	0.2125
0.2405	0.0915	0.1965	0.1555	0.1675
0.2325	0.0565	0.1525	0.1695	0.1755
0.2665	0.0485	0.1435	0.1615	0.1635
0.2725	0.05483333	0.15533333	0.138	0.184
98.8990826	16.6972477	61.1009174	54.4954128	65.8715596
112.844037	16.3302752	56.3302752	46.4220183	75.412844
116.880734	15.5963303	43.853211	24.4036697	77.9816514
88.2568807	33.5779817	72.1100917	57.0642202	61.4678899
85.3211009	20.733945	55.9633028	62.2018349	64.4036697
97.7981651	17.7981651	52.6605505	59.266055	60
100	20.1223242	57.0030581	50.6422018	67.5229358

Blank
0.056
0.063
0.064
0.077
0.07
0.068
0.06633333

SFM	Colistin 1200	Pio 100	Pio 150	Pio 200
0.311	0.092	0.123	0.181	0.241
0.262	0.1	0.153	0.19	0.178
0.249	0.1	0.127	0.215	0.212
0.287	0.107	0.165	0.211	0.23
0.24	0.077	0.157	0.161	0.184
0.248	0.111	0.131	0.159	0.189

0.24466667	0.02566667	0.05666667	0.11466667	0.17466667
0.19566667	0.03366667	0.08666667	0.12366667	0.11166667
0.18266667	0.03366667	0.06066667	0.14866667	0.14566667
0.22066667	0.04066667	0.09866667	0.14466667	0.16366667
0.17366667	0.01066667	0.09066667	0.09466667	0.11766667
0.18166667	0.04466667	0.06466667	0.09266667	0.12266667
0.19983334	0.0315	0.07633334	0.11983334	0.13933334
122.43536	12.8440379	28.3569649	57.3811507	87.4061706
97.9149275	16.8473739	43.3694748	61.8849037	55.8798997
91.4095065	16.8473739	30.3586328	74.3953286	72.8940776
110.425352	20.3502929	49.3744788	72.3936606	81.9015836
86.9057536	5.33778297	45.3711428	47.3728108	58.8824017
90.9090895	22.3519609	32.3603008	46.3719768	61.3844867
99.9999983	15.7631371	38.1984991	59.9666385	69.72477

Blank
0.049
0.055
0.059
0.066
0.074
0.066
0.0615

SFM	Colistin 1200	Pio 100	Pio 150	Pio 200
0.333	0.113	0.227	0.216	0.226
0.352	0.109	0.21	0.185	0.218
0.388	0.112	0.195	0.134	0.206
0.286	0.131	0.263	0.229	0.243
0.282	0.116	0.221	0.305	0.241
0.309	0.119	0.225	0.186	0.204

0.2715	0.0515	0.1655	0.1545	0.1645
0.2905	0.0475	0.1485	0.1235	0.1565
0.3265	0.0505	0.1335	0.0725	0.1445
0.2245	0.0695	0.2015	0.1675	0.1815
0.2205	0.0545	0.1595	0.2435	0.1795
0.2475	0.0575	0.1635	0.1245	0.1425
0.2635	0.05516667	0.162	0.14766667	0.1615
103.036053	19.544592	62.8083491	58.6337761	62.4288425
110.246679	18.0265655	56.3567362	46.8690702	59.3927894
123.908918	19.1650854	50.6641366	27.5142315	54.8387097
85.199241	26.3757116	76.4705882	63.5673624	68.8804554
83.6812144	20.683112	60.5313093	92.4098672	68.1214421
93.9278937	21.8216319	62.0493359	47.2485769	54.0796964
100	20.9361164	61.4800759	56.0404807	61.2903226

Experiment 1

Sample Name	Target Name	Cт	Cт Mean	ΔCт Mean	ΔΔCт
CONTROL	GAPDH	18.9729614			
CONTROL	GAPDH	18.7619667			
CONTROL	GAPDH	18.8933926	18.8761069		
COLISTIN	GAPDH	18.9320698			
COLISTIN	GAPDH	18.9825706			
COLISTIN	GAPDH	18.9778538	18.9641647		
PIOGLITAZONE	GAPDH	18.8215237			
PIOGLITAZONE	GAPDH	18.7138729			
PIOGLITAZONE	GAPDH	18.7213211	18.7522392		
COLISTIN+PIOGLITAZO	GAPDH	18.6028061			
COLISTIN+PIOGLITAZO	GAPDH	18.7376232			
COLISTIN+PIOGLITAZO	GAPDH	18.7209702	18.6871332		
CONTROL	IL6	22.9370079			
CONTROL	IL6	23.10145			
CONTROL	IL6	22.2084923	22.7489834	3.87287649	0
COLISTIN	IL6	21.0342751			
COLISTIN	IL6	21.8904991			
COLISTIN	IL6	22.0874195	21.6707312	2.70656649	-1.16631
PIOGLITAZONE	IL6	23.5952759			
PIOGLITAZONE	IL6	23.4321346			
PIOGLITAZONE	IL6	22.9130878	23.3134995	4.56126022	0.68838374
COLISTIN+PIOGLITAZO	IL6	22.3641968			
COLISTIN+PIOGLITAZO	IL6	21.4979992			
COLISTIN+PIOGLITAZO	IL6	21.0519676	21.6380545	2.95092138	-0.9219551

Experiment 2

Sample Name	Target Name	Cт	Cт Mean	ΔCт Mean	ΔΔCт
CONTROL	GAPDH	17.5905361			
CONTROL	GAPDH	17.5360451			
CONTROL	GAPDH	17.9317856	17.686121		
COLISTIN	GAPDH	18.665741			
COLISTIN	GAPDH	19.405941			
COLISTIN	GAPDH	19.8458347	19.3058395		
PIOGLITAZONE	GAPDH	17.537138			
PIOGLITAZONE	GAPDH	17.6469975			
PIOGLITAZONE	GAPDH	17.8809566	17.688364		
COLISTIN+PIOGLITAZONE	GAPDH	17.6924362			
COLISTIN+PIOGLITAZONE	GAPDH	18.035984			
COLISTIN+PIOGLITAZONE	GAPDH	18.1472416	17.9585539		

CONTROL	IL6	20.6284218			
CONTROL	IL6	19.8800316			
CONTROL	IL6	20.5978088	20.3687541	2.68263308	0
COLISTIN	IL6	20.9535313			
COLISTIN	IL6	20.8759289			
COLISTIN	IL6	21.4323559	20.9147301	1.60889053	-1.0737425
PIOGLITAZONE	IL6	20.9662495			
PIOGLITAZONE	IL6	21.2808361			
PIOGLITAZONE	IL6	20.8925896	21.0465565	3.35819244	0.67555936
COLISTIN+PIOGLITAZONE	IL6	20.8769855			
COLISTIN+PIOGLITAZONE	IL6	20.6166077			
COLISTIN+PIOGLITAZONE	IL6	19.6868019	20.393465	2.43491109	-0.247722

Experiment 3

Sample Name	Target Name	CT	CT Mean	ΔCT Mean	ΔΔCT
CONTROL	GAPDH	18.6377888			
CONTROL	GAPDH	18.6271973			
CONTROL	GAPDH	18.7386112	18.6678658		
COLISTIN	GAPDH	18.7298756			
COLISTIN	GAPDH	18.937273			
COLISTIN	GAPDH	18.9196301	18.8622595		
PIOGLITAZONE	GAPDH	18.3623962			
PIOGLITAZONE	GAPDH	18.4261456			
PIOGLITAZONE	GAPDH	18.5009079	18.4298166		
COLISTIN+PIOGLITAZO	GAPDH	18.4545403			
COLISTIN+PIOGLITAZO	GAPDH	18.3781567			
COLISTIN+PIOGLITAZO	GAPDH	18.3848305	18.4058425		
CONTROL	IL6	22.4492569			
CONTROL	IL6	22.7325134			
CONTROL	IL6	22.8792439	22.6870047	4.01913897	0
COLISTIN	IL6	21.4214439			
COLISTIN	IL6	21.4548855			
COLISTIN	IL6	20.6240883	21.1668059	2.30454636	-1.7145926
PIOGLITAZONE	IL6	23.649229			
PIOGLITAZONE	IL6	23.2418156			
PIOGLITAZONE	IL6	23.093483	23.3281759	4.8983593	0.87922033
COLISTIN+PIOGLITAZO	IL6	21.808794			
COLISTIN+PIOGLITAZO	IL6	21.6171017			
COLISTIN+PIOGLITAZO	IL6	21.7353516	21.7204158	3.31457329	-0.7045657

Fold change

1

2.24436916

0.62054867

1.89468118

Fold change

1

2.10488666 pipette error

0.62608942

1.18733084

Fold change

1

3.28203953

0.54366116

1.62965399

Experiment 1

Sample Name	Target Name	CT	CT Mean	ΔCT Mean	ΔΔCT
CONTROL	GAPDH	18.9729614			
CONTROL	GAPDH	18.7619667			
CONTROL	GAPDH	18.8933926	18.8761069		
COLISTIN	GAPDH	18.9320698			
COLISTIN	GAPDH	18.9825706			
COLISTIN	GAPDH	18.9778538	18.9641647		
PIOGLITAZONE	GAPDH	18.8215237			
PIOGLITAZONE	GAPDH	18.7138729			
PIOGLITAZONE	GAPDH	18.7213211	18.7522392		
COLISTIN+PIOGLITAZON	GAPDH	18.6028061			
COLISTIN+PIOGLITAZON	GAPDH	18.7376232			
COLISTIN+PIOGLITAZON	GAPDH	18.7209702	18.6871332		
CONTROL	TNFA	30.8138561			
CONTROL	TNFA	30.9263821			
CONTROL	TNFA	30.8778305	30.8726902	11.9965833	0
COLISTIN	TNFA	29.713068			
COLISTIN	TNFA	29.2030296			
COLISTIN	TNFA	29.3198509	29.4119816	10.4478168	-1.5487665
PIOGLITAZONE	TNFA	30.8698921			
PIOGLITAZONE	TNFA	30.8078995			
PIOGLITAZONE	TNFA	30.9757595	30.8845158	12.1322765	0.13569323
COLISTIN+PIOGLITAZON	TNFA	29.7645531			
COLISTIN+PIOGLITAZON	TNFA	29.9231853			
COLISTIN+PIOGLITAZON	TNFA	30.1352673	29.9410019	11.2538687	-0.7427146

Sample Name	Target Name	CT	CT Mean	ΔCT Mean	ΔΔCT
CONTROL	GAPDH	17.9969864			
CONTROL	GAPDH	17.9918728			
CONTROL	GAPDH	17.9423103	17.9770565		
COLISTIN	GAPDH	17.9900322			
COLISTIN	GAPDH	17.9255791			
COLISTIN	GAPDH	17.9504147	17.955342		
PIOGLITAZONE	GAPDH	17.9449272			
PIOGLITAZONE	GAPDH	17.9594269			
PIOGLITAZONE	GAPDH	17.8851089	17.929821		
COLISTIN+PIOGLITAZON	GAPDH	17.8323631			
COLISTIN+PIOGLITAZON	GAPDH	17.8825092			
COLISTIN+PIOGLITAZON	GAPDH	17.8618679	17.8589134		
CONTROL	TNFA	30.8130379			

CONTROL	TNFA	30.6408787			
CONTROL	TNFA	30.3337498	30.5958888	12.6188323	0
COLISTIN	TNFA	29.4375973			
COLISTIN	TNFA	29.3703003			
COLISTIN	TNFA	29.1089935	29.3056304	11.3502884	-1.2685439
PIOGLITAZONE	TNFA	30.9050865			
PIOGLITAZONE	TNFA	30.1912613			
PIOGLITAZONE	TNFA	30.3928051	30.4963843	12.5665633	-0.052269
COLISTIN+PIOGLITAZONI	TNFA	29.9331779			
COLISTIN+PIOGLITAZONI	TNFA	29.4170856			
COLISTIN+PIOGLITAZONI	TNFA	29.9093475	29.7532037	11.8942903	-0.724542

Sample Name	Target Name	CT	CT Mean	ΔCT Mean	ΔΔCT
CONTROL	GAPDH	18.6377888			
CONTROL	GAPDH	18.6271973			
CONTROL	GAPDH	18.7386112	18.6678658		
COLISTIN	GAPDH	18.7298756			
COLISTIN	GAPDH	18.937273			
COLISTIN	GAPDH	18.9196301	18.8622595		
PIOGLITAZONE	GAPDH	18.3623962			
PIOGLITAZONE	GAPDH	18.4261456			
PIOGLITAZONE	GAPDH	18.5009079	18.4298166		
COLISTIN+PIOGLITAZONI	GAPDH	18.4545403			
COLISTIN+PIOGLITAZONI	GAPDH	18.3781567			
COLISTIN+PIOGLITAZONI	GAPDH	18.3848305	18.4058425		
CONTROL	TNFA	31.3848324			
CONTROL	TNFA	31.2572613			
CONTROL	TNFA	31.2659016	31.3026651	12.6347993	0
COLISTIN	TNFA	29.8788109			
COLISTIN	TNFA	29.9057121			
COLISTIN	TNFA	29.8534317	29.8793182	11.0170587	-1.6177406
PIOGLITAZONE	TNFA	31.0093803			
PIOGLITAZONE	TNFA	30.9246044			
PIOGLITAZONE	TNFA	30.8835468	30.9391772	12.5093606	-0.1254387
COLISTIN+PIOGLITAZONI	TNFA	32.8270302	(OUTLIER DUE TO PIPETTING ERROR!)		
COLISTIN+PIOGLITAZONI	TNFA	30.7562714			
COLISTIN+PIOGLITAZONI	TNFA	30.9722137	30.8642426	12.4584001	-0.1763992

Experiment 2

Fold change

1

2.92566879

0.91023235

1.67332139

Fold change

1

2.40918283

1.03689441

1.65237597

Fold change

1

3.06894041

1.09083938

1.13005989

Experiment 3

GEL 1	NFKB	Beta actin	Normalized NFKB Expressio
Control	33681.702	33704.066	0.99933646
Pioglitazone	41829.945	30728.187	1.361289067
Colistin	29962.167	21884.995	1.369073514
Colistin + Pioglitazone	25012.823	27095.53	0.923134665

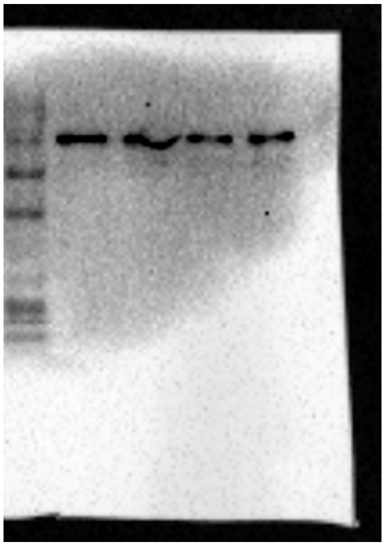

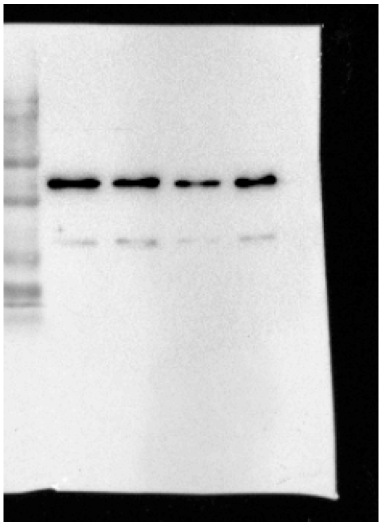

Fold Change Relative to Control	GEL 2	NFKB	Beta actin
1	Control	40092.652	27183.551
1.362192936	Pioglitazone	37308.702	33739.652
1.369982552	Colistin	24888.066	9463.359
0.923747608	Colistin + Pioglitazone	26739.167	35471.48
	Control	23358.581	32774.924
	Pioglitazone	18711.53	32395.773
	Colistin	33816.087	23758.087
	Colistin + Pioglitazone	8207.995	12364.137

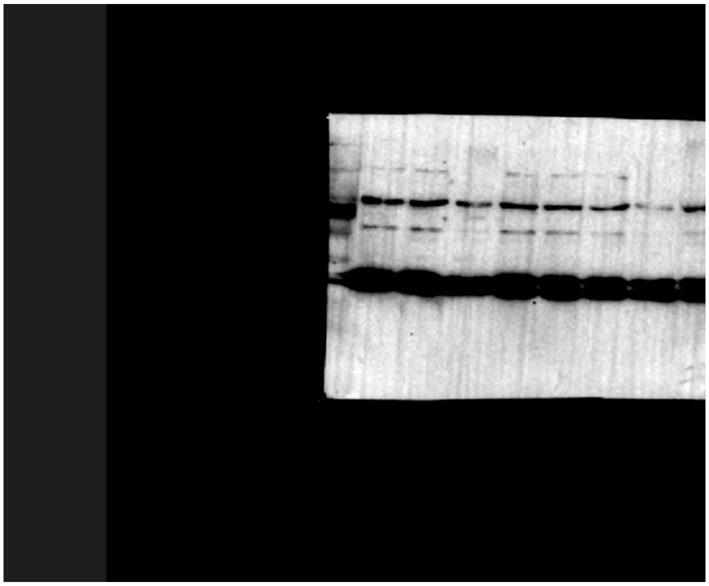

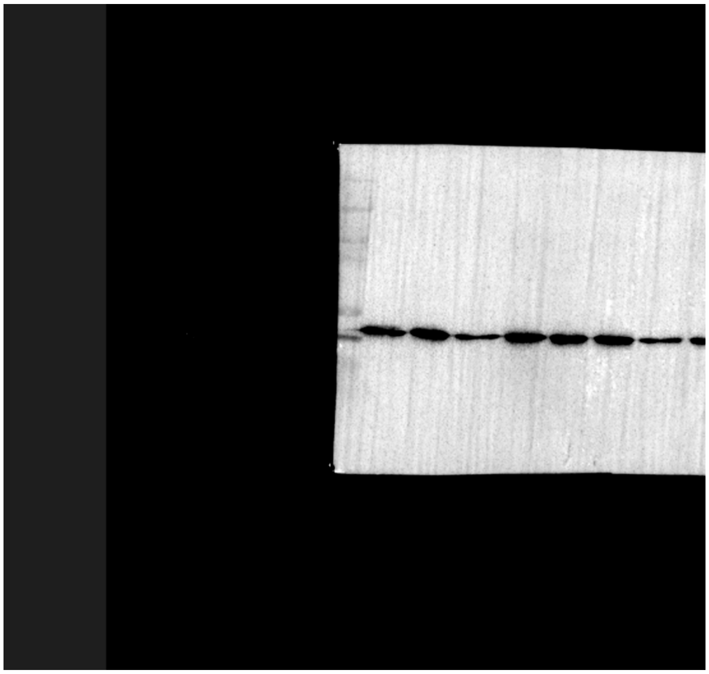

Normalized NFKB Expressior	Fold Change Relative to Control
1.474886486	0.999999997
1.105782063	0.749740452
2.629939961	1.783147367
0.753821577	0.511104809

0.712696725	1
0.577591712	0.810431271
1.423350584	1.997133611
0.663855067	0.931469227

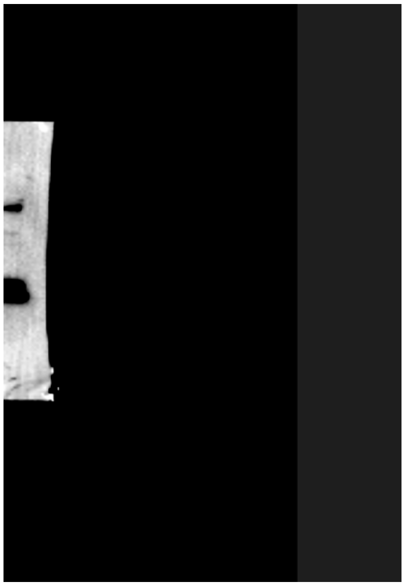

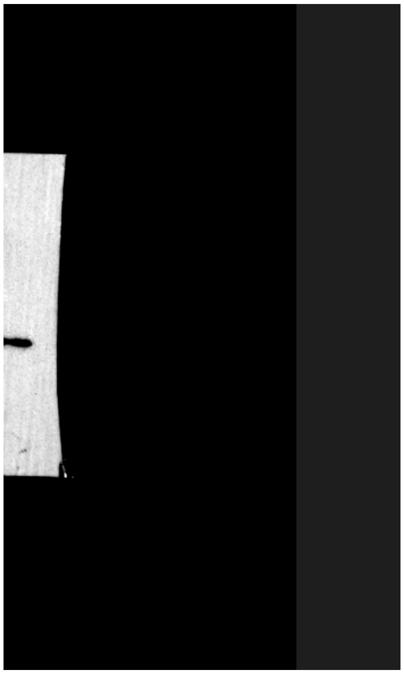

1	1.36219294	1.36998255	0.92374761
1	0.74974045	1.78314737	0.51110481
1	0.81043127	1.99713361	0.93146923

GEL 1	NRF2	GAPDH	Normalized NRF2 Expression
Control	30624.652	37747.681	0.811298898
Pioglitazone	28682.116	32912.652	0.87146171
Colistin	12484.309	27185.187	0.459232044
Colistin + Pioglitazone	27199.38	23975.288	1.134475632

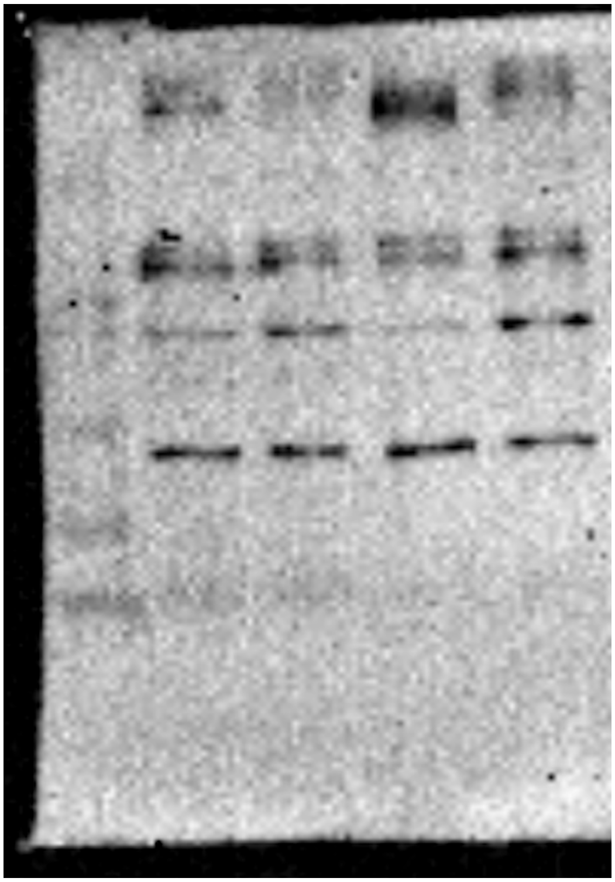

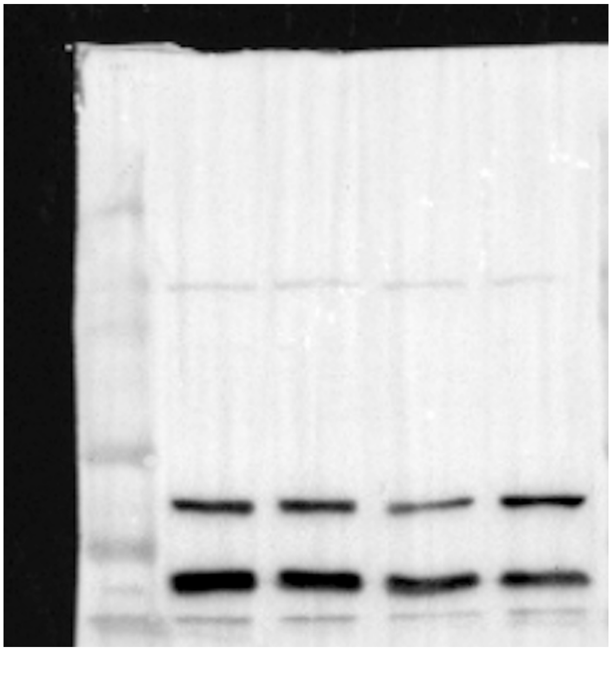

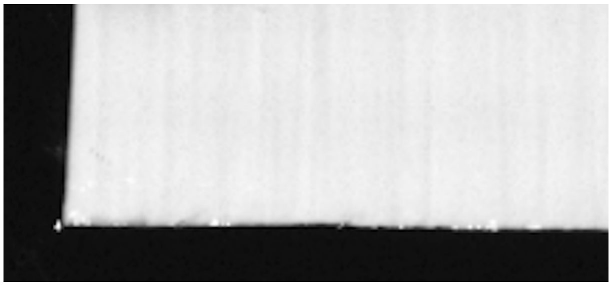

Fold Change Relative to Control
0.999999998
1.074156159
0.566045442
1.398344842

GEL 2
Control
Pioglitazone
Colistin
Colistin + Pioglitazone

Control
Pioglitazone
Colistin
Colistin + Pioglitazone

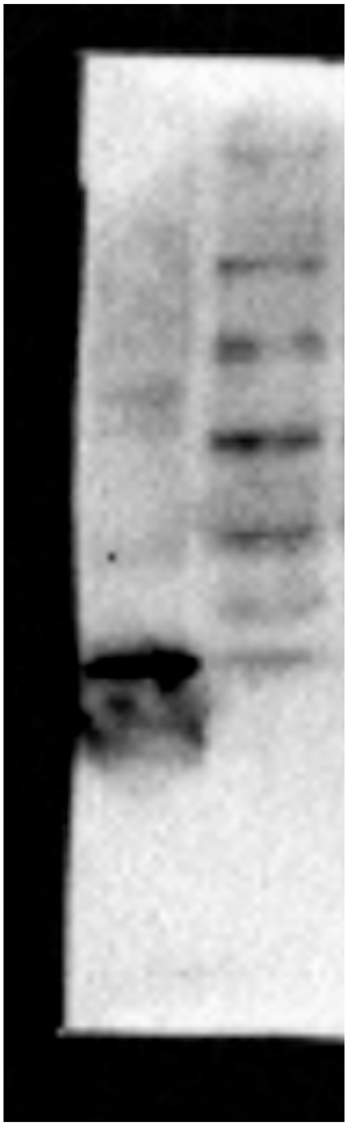

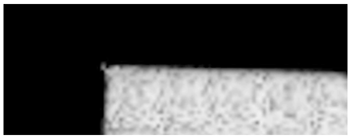

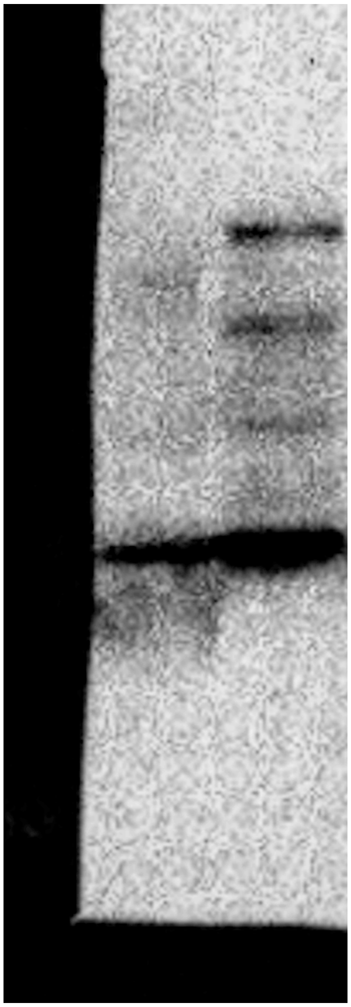

NRF2	GAPDH	Normalized N
31824.359	40063.359	0.79435074
33241.187	31287.773	1.06243378
3190.803	17463.823	0.18270931
33250.945	35812.409	0.92847552
30430.116	44300.459	0.68690295
30209.924	36893.581	0.81883957
12164.752	30944.016	0.39312131
19469.116	36250.48	0.53707195

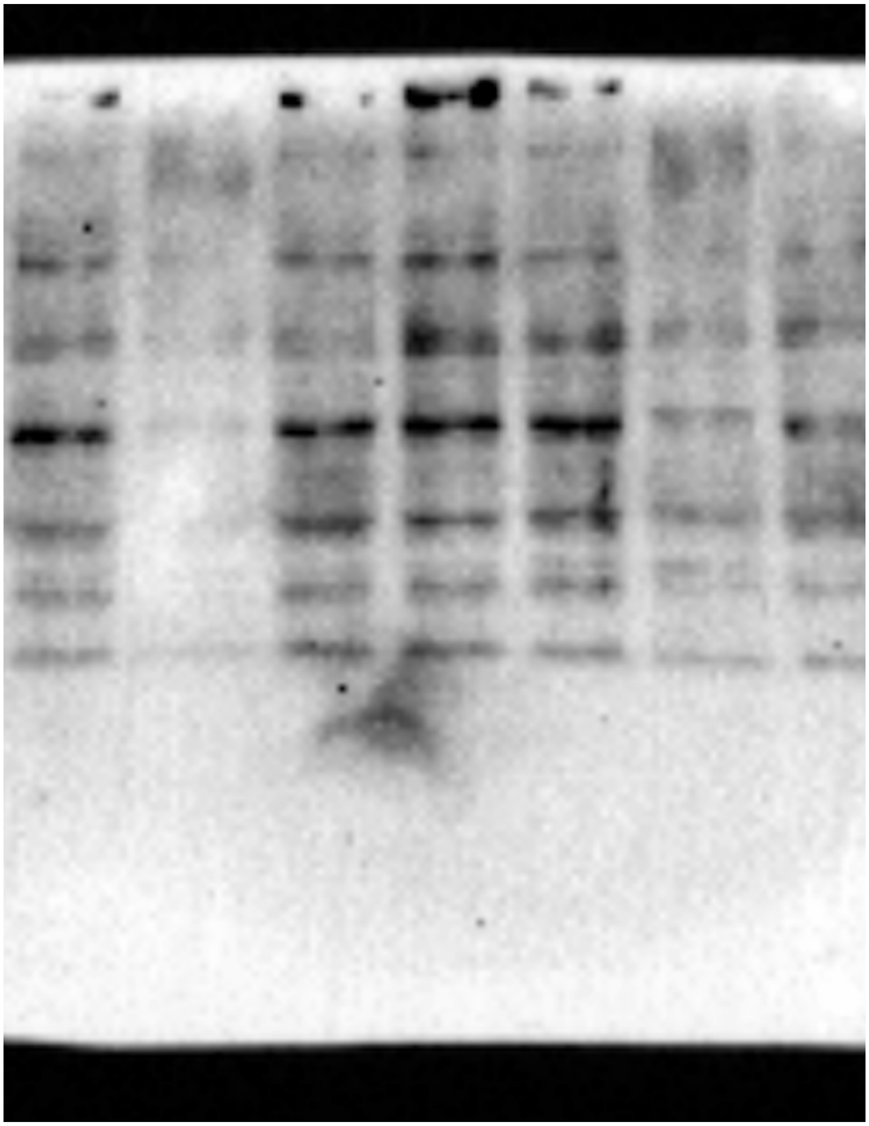

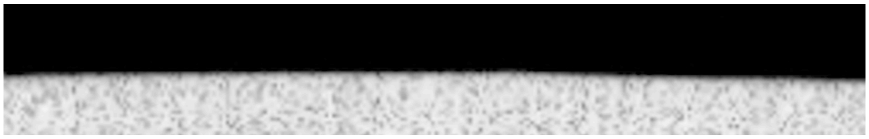

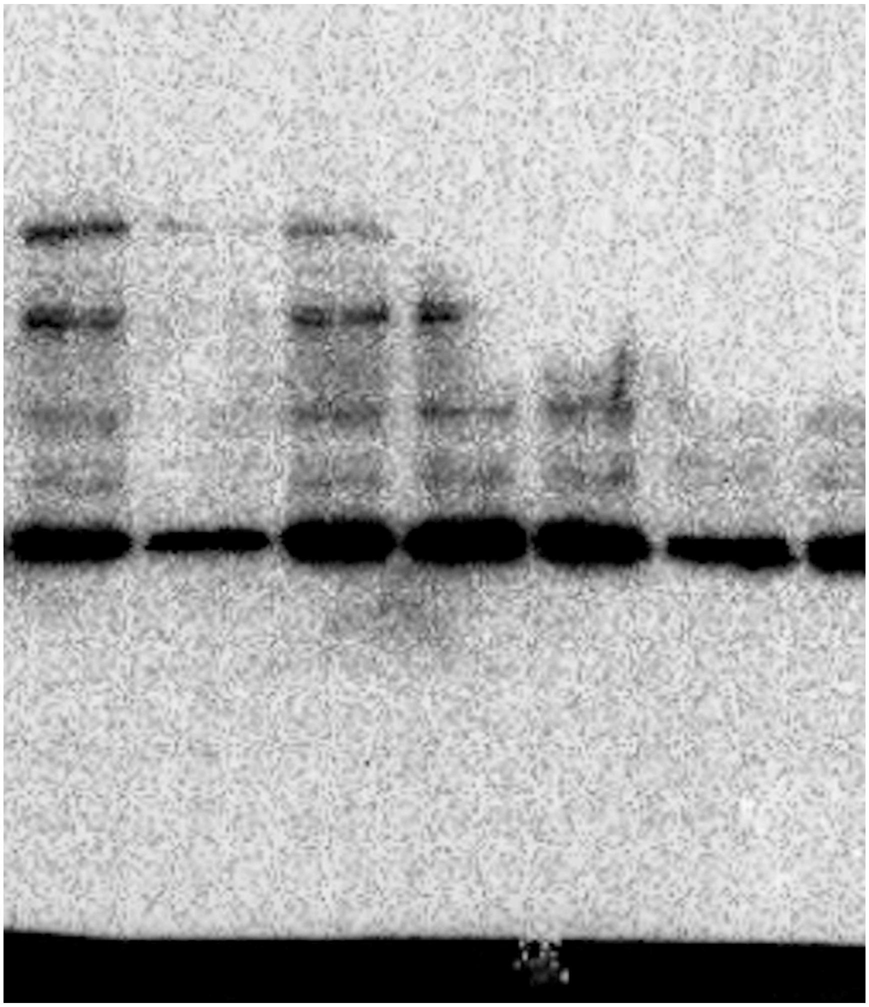

Fold Change Relative to Control
1.000000004
1.337486992
0.230010873
1.168848308

1.000000001
1.192074615
0.572309824
0.781874573

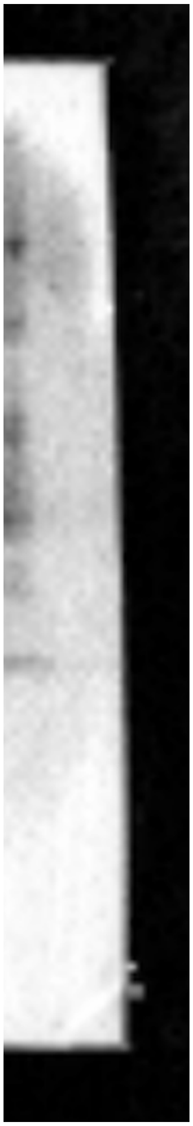

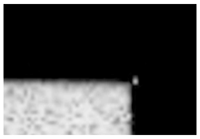

Supplement: S1 Raw images — (PDF) [file pone.0314092.s002.pdf]
