# Supplementary material for: The ameliorative effect of pioglitazone against colistin-induced nephrotoxicity is mediated by inhibition of NF-κB and restoration of Nrf2 signaling: An integrative bioinformatics prediction-guided in vitro study
Source: PLoS One. 2024 Dec 2;19(12):e0314092. doi: 10.1371/journal.pone.0314092 (PMC11611169; doi:10.1371/journal.pone.0314092)
Supplement: Attachment — Submitted filename: Review comments.docx [file pone.0314092.s003.docx]

Dear Authors,

Congratulations on your significant findings. The work holds considerable value as it provides potential insights into effectively managing colistin-induced toxicity. However, to enhance the clarity and depth of your study, several nuanced areas require further attention:

1. Beyond nephrotoxicity, the NF-κB and Nrf2 pathways are involved in various cellular processes. Did your study demonstrate that the reported anti-inflammatory and antioxidative effects of Pioglitazone are specific to colistin-induced toxicity, or could these effects be general responses to other nephrotoxic agents? Were appropriate controls used to distinguish between specific and non-specific protective effects? To strengthen the study and confirm specificity, employing an inhibitor is recommended.
2. Given the interconnected nature of these pathways, Pioglitazone may directly or indirectly modulate them. Are there additional mechanisms, aside from those reported, that could contribute to Pioglitazone's nephroprotective effects? Were these potential mechanisms considered in your study?
3. Considering the implications of long-term Pioglitazone use, could chronic administration introduce complications not evident within the study's timeframe?
4. Additionally, with the growing concern over colistin resistance, Pioglitazone's nephroprotective effects might inadvertently encourage the continued use of colistin, potentially exacerbating antibiotic resistance issues. Could you discuss the broader implications of promoting Pioglitazone in this context?
5. Lastly, when extrapolating your findings, it is important to consider that Pioglitazone’s activation of PPARγ may have off-target effects, such as fluid retention or cardiovascular complications, which could counterbalance its nephroprotective benefits. Does your study account for these potential off-target effects? Addressing this is crucial, as any detrimental effects could outweigh the overall benefits of the drug.

These points should be addressed to improve the study's overall clarity and robustness.
