# Supplementary material for: The ameliorative effect of pioglitazone against colistin-induced nephrotoxicity is mediated by inhibition of NF-κB and restoration of Nrf2 signaling: An integrative bioinformatics prediction-guided in vitro study
Source: PLoS One. 2024 Dec 2;19(12):e0314092. doi: 10.1371/journal.pone.0314092 (PMC11611169; doi:10.1371/journal.pone.0314092)
Supplement: Attachment — Submitted filename: Response to Reviewer Comments Manuscript PONE-D-24-32686.docx [file pone.0314092.s004.docx]

**Response to PLOS ONE Reviewers for Manuscript PONE-D-24-32686**

**Reviewer #1: Peer Review Report for Manuscript PONE-D-24-32686**

**First of all, we would like to thanks the reviewers for their time and valuable comments regarding the manuscript. We have answered all the comments and highlighted them in the main manuscript.**

Summary of the Research and Overall Impression

The manuscript titled "The Ameliorative Effect of Pioglitazone Against Colistin-induced Nephrotoxicity is Mediated by Inhibition of NF-κB and Restoration of Nrf2 Signaling: An Integrative Bioinformatics Prediction-Guided In Vitro Study" investigates the protective effects of pioglitazone against colistin-induced nephrotoxicity. The study employs a combination of bioinformatics predictions and in vitro experiments using HK-2 human kidney cells to explore the underlying mechanisms. The authors demonstrate that pioglitazone modulates NF-κB-mediated inflammatory signaling and Nrf2-mediated antioxidative stress pathways, leading to increased cell viability and reduced lactate dehydrogenase release in colistin-treated cells.

Overall, this study provides valuable insights into the potential therapeutic application of pioglitazone in preventing colistin-induced kidney damage. The integrative approach combining bioinformatics and experimental validation is a significant strength of this manuscript. However, several methodological and interpretative issues need to be addressed to strengthen the validity and impact of the findings.

Recommendation: Major Revision

Major Issues

1. Lack of Controls in Bioinformatics Predictions:

o The manuscript does not include appropriate controls in the bioinformatics analysis to ensure specificity. Including negative controls, such as unrelated drugs or conditions, would strengthen the claim that the observed effects are specific to the pioglitazone and colistin interaction (Introduction, Methodology).

**Answer 1:** We thank the reviewer for the very valuable comment about the need for appropriate controls in our bioinformatics analysis to ensure specificity. While this is a limitation in our study, we have witnessed prior studies that have undertaken a similar bioinformatics approach without the inclusion of controls (for example: <https://doi.org/10.1186/s12967-023-04233-0>). In this study, the authors intersected resveratrol-related genes with genes attributed to diabetic kidney disease without including any controls in their bioinformatics analysis in a manner similar to the approach we have undertaken in our study. We have also introduced two new figures (Figure 1a and 1b) to illustrate how we obtained the intersection genes and to classify the intersection genes to increase clarity of our bioinformatics study. In addition, we have validated the bioinformatics results in HK-2 cells to further strengthen the bioinformatics findings in our study. (Line 295-311)

2. Sample Size Justification:

o The manuscript lacks a clear justification for the sample sizes used in the in vitro experiments. A power analysis or rationale for the chosen sample sizes should be provided to ensure the statistical reliability of the findings (Methodology, Statistical Analysis).

**Answer 2:** We thank the reviewer for the very important comment on the statistical analysis employed in our study. We agree that we should have done a power analysis for the chosen sample size as that would have affected the statistical significance of the result. This is why we have redone our statistics for all of the in vitro studies included in our manuscript and we removed results that may have been compromised due to the lack of power analysis. We have rechecked the results of the newly reported data and we ensured the validity of our statistical analysis through checking the normality of the data using the Shapiro-wilk test and we also ensured the equality of the variance using the modified Levane test (Brown-Forsythe test) this ensured the validity of our one-way ANOVA and ensuring the statistical reliability of the findings. (line 285-291)

3. Potential Overinterpretation of Findings:

o The manuscript suggests that pioglitazone restores Nrf2 signaling solely based on in vitro data. While promising, these findings should be presented more cautiously, acknowledging the need for further validation in in vivo models (Discussion).

**Answer 3:** We thank the reviewer for the very important comment on our overinterpertation of the effect of pioglitazone on Nrf2 signaling. We absolutely agree that in vivo studies are required to establish the effect of pioglitazone on Nrf2 signaling in the context of colistin-induced nephrotoxicity. We have noted that our study requires extra validation in in vivo models to increase its translational value as a pre-clinical study in the last paragraph of the discussion section. We intend to undertake in vivo studies to confirm our in silico and in vitro findings in the future.

4. Lack of Ethical Approval Statement:

o Although the study uses human cell lines, there is no mention of ethical approval for these experiments. A statement regarding ethical approval or exemption should be included to clarify compliance with ethical standards (Ethical Considerations).

**Answer 4:** We thank the reviewer for reminding us that even when using cell lines, which do not require an ethnical approval, an ethical exemption statement is required to be included in the manuscript. We have added that to the revised manuscript. (Line 606)

Minor Issues

1. Redundancy and Conciseness:

o The manuscript contains redundant phrases, such as repeatedly stating "the ameliorative effect of pioglitazone against colistin-induced nephrotoxicity." The authors should streamline the text to improve readability (Introduction, Discussion).

**Answer 1**: We agree with the reviewer that removing the overused phrase “the ameliorative effect of pioglitazone against colistin-induced nephrotoxicity.” in our manuscript will improve its readability. Hence, we have removed some of these redundant phrases to improve the readability of our introduction and discussion sections. (Line 157-158), (Line 526), and (Line 574-575)

2. Comprehensiveness of Literature Review:

o The literature review could be expanded to include more recent studies on colistin-induced nephrotoxicity and Nrf2 signaling in renal protection. This would provide a more comprehensive context for the study's findings (Introduction).

**Answer 2**: We thank the reviewer for this important comment and we acknowledge that we have not provided enough literature review in the introduction section, particularly pertaining to the role of Nrf2 signaling in the context of colistin-induced nephrotoxicity. This is why we have revamped our introduction section and included recent studies that have studied the role of Nrf2 signaling in the context of colistin-induced nephrotoxicity. (Line 120-131)

3. Availability of Raw Data:

o The manuscript does not mention the availability of raw data or supplementary materials. The authors should consider providing access to raw data and supplementary materials to enhance reproducibility (Methodology, Results).

**Answer 3:** We thank the reviewer for suggesting access to the raw data underlying the findings of this study to improve confidence in our results and enhance the reproducibility of the study. We have provided the raw data in the form of an excel sheet that contains clearly labelled sections related to the in vitro experiments in our manuscript. In addition, we have also submitted a supplementary file containing all of the bioinformatics data we used and obtained in this study. The supplementary file sections are mentioned in the results section to enhance it and provide all of the data that could not be incorporated in the manuscript to avoid overcomplicating the manuscript for the respected readers. (Raw data is available in excel sheet titled Raw Data Plos One)

Any Other Points

Confidential Comments to the Editors: The study's overall approach is sound, but the manuscript requires revisions to address improved clarity. There are no ethical concerns related to this study.

**Reviewer #2: Peer Review Report for Manuscript PONE-D-24-32686**

Dear Authors,

Congratulations on your significant findings. The work holds considerable value as it provides potential insights into effectively managing colistin-induced toxicity. However, to enhance the clarity and depth of your study, several nuanced areas require further attention:

1. Beyond nephrotoxicity, the NF-κB and Nrf2 pathways are involved in various cellular processes. Did your study demonstrate that the reported anti-inflammatory and antioxidative effects of Pioglitazone are specific to colistin-induced toxicity, or could these effects be general responses to other nephrotoxic agents? Were appropriate controls used to distinguish between specific and non-specific protective effects? To strengthen the study and confirm specificity, employing an inhibitor is recommended.

**Answer 1**: We thank the reviewer for this very important and critical comment regarding the specificity of our findings on pioglitazone protection against colistin-induced nephrotoxicity. We think that the effect of pioglitazone on colistin-induced nephrotoxicity is not specific to just colistin but can be interpreted as a general response to oxidative stress and inflammation that accompany acute kidney injury (AKI) in renal proximal tubular epithelial cells. Pioglitazone has previously shown protective effects in cisplatin-induced nephrotoxicity and other models of AKI like the ischemia-reperfusion injury model (<https://doi.org/10.1111/jcmm.15782>)(<https://doi.org/10.1016/j.fct.2012.09.006>) (<https://doi.org/10.1016/j.bcp.2015.11.027>)([10.3389/fphar.2018.00851](https://doi.org/10.3389%2Ffphar.2018.00851))(<https://doi.org/10.1016/j.jss.2012.01.012>). We do agree that we should have used a PPAR-γ inhibitor such as GW9662 to further strenghtnen the mechanistic findings of our study. However, our goal was not to study the mechanistics in detail, but rather we sought to confirm the validity of the hypothesis generated through the bioinformatics study. We intend to delve deeper into the mechanistics and specific v.s. non-specific effects in the in vivo study that we intend to do in the future. In that study, we will surely use GW9662 in one of the groups to discern whether the protective effect of pioglitazone is PPAR-γ specific or not.

2. Given the interconnected nature of these pathways, Pioglitazone may directly or indirectly modulate them. Are there additional mechanisms, aside from those reported, that could contribute to Pioglitazone's nephroprotective effects? Were these potential mechanisms considered in your study?

**Answer 2:** Yes there are other mechanisms aside from NF-κB and Nrf2 pathways reported in this study that may underlie the protective effect of pioglitazone in the context of colistin-induced nephrotoxicity. We have undertaken a bioinformatics study in a rat microarray model of colistin-induced nephrotoxicity (unpublished data that is going to be published soon) and we found that in addition to the abovementioned pathways, pioglitazone increases adaptive dedifferentiation in proximal tubular epithelial cells through increasing macrophage polarization to the reparative M2 phenotype and reducing apoptsis of HK-2 cells. We conclude that in an animal model pioglitazone treatment increases adaptive repair of renal proximal tubular epithelial cells and thwarts the emergence of fibrosis through its effect on macrophage polarization.

3. Considering the implications of long-term Pioglitazone use, could chronic administration introduce complications not evident within the study's timeframe?

**Answer 3:** We thank the reviewer for this comment that aims to enhance the translational value of our study. Our study did not address any complications that may arise from the chronic administration of pioglitazone. We know from the clinical use of pioglitazone that it is associated with adverse effects that include water retention and exacerbation of heart failure and in some extreme cases it might be associated with increased risk of bladder cancer. However, the goal of our pre-clinical study was just to prove the protective effect of pioglitazone and identify pathways that could be responsible for it. We agree that our study requires to be confirmed in animal models and even in clinical trials. However, we envision that the translational value of this study lies in the continued use of pioglitazone in diabetic patients who are already stable on it prior to admission to the intensive care unit and requiring treatment with colistin. We think that the continued use of pioglitazone in these patient populations compared to transitioning to insulin may offer greater clinical value in the context of colistin mediated kidney toxicity. We agree that this requires to be validated in large clinical trials and hopefully if we could undertake several in vivo studies we could translate our findings to clinical trials in the future.

4. Additionally, with the growing concern over colistin resistance, Pioglitazone's nephroprotective effects might inadvertently encourage the continued use of colistin, potentially exacerbating antibiotic resistance issues. Could you discuss the broader implications of promoting Pioglitazone in this context?

**Answer 4:** We thank our respected reviewer for raising the important issue of antibiotic misuse and resistance that is considered a critical issue nowadays. Sadly, colistin resistance is on the rise in China and in other places around the world aswell. With the drying pipeline of newly introduced antibiotics and the high prevalence of antibiotic resistance, patients suffering from multi-drug resistant gram negative bacterial infections are at great risk of mortality. Therefore, antibiotics like colistin that is considered a last line option are important and have to be safeguarded from bacterial resistance. We think that pioglitazone may actually reduce the emergence of bacterial resistance as it has been shown to impove bacterial sensitivity to other antibiotics and it improves the immune system anti-bacterial effect. For example: A study by Masadeh et al., reported that pioglitazone induced a dose-dependent antibacterial activity against Escherichia Coli at a concentration of 80 micromolar (<https://doi.org/10.2147/DDDT.S24126>). Moreover, pre-treatment of bacteria with pioglitazone at a suboptimal concentration of 40 micromolar increased the antimicrobial activity of amoxicillin, cephalexin, and ciprofloxacin against a wide range of bacterial species, especially pseudomonas aeruginosa. In another study, administration of pioglitazone to burned mice 3 hours before subjection to post-burn infection with Escherichia Coli rescued them from fatal septic shock and significantly increased survival rates compared to vehicle-treated mice (<https://doi.org/10.3390/ijms232112746>). Pioglitazone pre-treatment lowered the hepatic and plasma bacterial load and significantly reduced hepatic degeneration and lowered alanine transaminase levels in the blood. The authors concluded that pioglitazone through activation of PPAR-γ significantly reduced the peak in pro-inflammatory cytokines accompanying septic shock and increased the recruitment of CD11b^+^F4/80^low^ macrophages leading to increased phagocytosis and clearance of bacteria. Moreover, pioglitazone shifted liver macrophages to an M2-like phenotype thereby reducing inflammatory damage. These studies show that pioglitazone can complement the anti-bacterial effect of antibiotics, thereby reducing the ability of the bacteria to develop bacterial resistance that accompany failed treatment.

5. Lastly, when extrapolating your findings, it is important to consider that Pioglitazone’s activation of PPARγ may have off-target effects, such as fluid retention or cardiovascular complications, which could counterbalance its nephroprotective benefits. Does your study account for these potential off-target effects? Addressing this is crucial, as any detrimental effects could outweigh the overall benefits of the drug.

**Answer 5:** We thank the reviewer for pointing this out and we do agree that pioglitazone is not for everyone. Even for diabetics, pioglitazone is considered a second-choice drug compared to metformin or the sodium glucose cotransporter 2 inhibitors that are safer for the majority of patients. However, pioglitazone use in the context of colistin-induced kidney injury is reserved for those patients who were already stable on pioglitazone prior to contracting multi-drug resistant bacteria that requires admission to the intensive care unit and commencement of colistin therapy. As we said earlier we believe that continuing pioglitazone rather than or in addition to transitioning to insulin may provide a greater clinical value with regards to the development of colistin renal toxicity and even kidney adaptive repair response and resolution of kidney injury. However, we believe that this has to be proven in multiple clinical trials and several in vivo studies are required before transitioning to human clinical trials. Overall, we thank our respected reviewer for highlighting these critical points in our study that aim to improve its clarity. And we hope that we have appropriately alleviated concerns regarding the translational value of this study.
